# Case Fatality Ratio Estimates for the 2013–2016 West African Ebola Epidemic: Application of Boosted Regression Trees for Imputation

**DOI:** 10.1093/cid/ciz678

**Published:** 2019-07-22

**Authors:** Alpha Forna, Pierre Nouvellet, Ilaria Dorigatti, Christl A Donnelly

**Affiliations:** 1 Medical Research Council Centre for Global Infectious Disease Analysis, Department of Infectious Disease Epidemiology, Brighton, Brighton, United Kingdom, and Imperial College London, London, United Kingdom; 2 School of Life Sciences, University of Sussex, Brighton, Brighton, United Kingdom; 3 Department of Statistics, University of Oxford, Oxford, United Kingdom

**Keywords:** machine learning, survival, viral hemorrhagic disease, imputation, infectious disease epidemiology

## Abstract

**Background:**

The 2013–2016 West African Ebola epidemic has been the largest to date with >11 000 deaths in the affected countries. The data collected have provided more insight into the case fatality ratio (CFR) and how it varies with age and other characteristics. However, the accuracy and precision of the naive CFR remain limited because 44% of survival outcomes were unreported.

**Methods:**

Using a boosted regression tree model, we imputed survival outcomes (ie, survival or death) when unreported, corrected for model imperfection to estimate the CFR without imputation, with imputation, and adjusted with imputation. The method allowed us to further identify and explore relevant clinical and demographic predictors of the CFR.

**Results:**

The out-of-sample performance (95% confidence interval [CI]) of our model was good: sensitivity, 69.7% (52.5–75.6%); specificity, 69.8% (54.1–75.6%); percentage correctly classified, 69.9% (53.7–75.5%); and area under the receiver operating characteristic curve, 76.0% (56.8–82.1%). The adjusted CFR estimates (95% CI) for the 2013–2016 West African epidemic were 82.8% (45.6–85.6%) overall and 89.1% (40.8–91.6%), 65.6% (61.3–69.6%), and 79.2% (45.4–84.1%) for Sierra Leone, Guinea, and Liberia, respectively. We found that district, hospitalisation status, age, case classification, and quarter (date of case reporting aggregated at three-month intervals) explained 93.6% of the variance in the naive CFR.

**Conclusions:**

The adjusted CFR estimates improved the naive CFR estimates obtained without imputation and were more representative. Used in conjunction with other resources, adjusted estimates will inform public health contingency planning for future Ebola epidemics, and help better allocate resources and evaluate the effectiveness of future inventions.

## Teaser

Corrected for imperfect imputation, boosted regression trees generated more representative case fatality ratio estimates than could be obtained without imputation. These estimates, used in tandem with other resources, are essential for public health planning and evaluation in resource-poor settings.

Ebola virus disease (EVD), a hemorrhagic fever disease of zoonotic origin, has been detected in several outbreaks in African countries since it was first identified in 1976 [1]. While outbreaks have typically been of relatively limited size and confined to rural Central Africa, the 2013–2016 mostly urbanized epidemic in West Africa took the world by surprise and showed the potentially devastating impact of the Ebola virus [1].

The case fatality ratio (CFR), the proportion of EVD cases who died [2], remains a challenging quantity to estimate due to undetected cases, incomplete data on detected cases, and the lack of laboratory confirmation of probable and suspected cases. CFR estimates may vary because of unreported survival outcomes, time of case diagnosis, and the statistical methods used for estimation [3, 4]. For instance, 14 694 (44%) of the survival outcomes for confirmed, probable, and suspected cases are unreported in the dataset analyzed by the World Health Organization (WHO) Ebola Response Team [5]. Using clinical, laboratory, and field surveillance data, we imputed unreported outcomes based on models fitted to observed outcome data. Imputed outcomes have previously been used in other contexts to generate baseline estimates of CFR necessary in evaluating interventions and designing randomized controlled trials [6].

Regression modeling has been used widely to predict EVD survival outcomes [7–9]. For instance, after the first 9 months of this epidemic, logistic regression was used to identify predictors of CFR [9]. It was found that age, sex, country, and fever were correlated with CFR [8]. However, much of the observed heterogeneity remained unaccounted for. Thus, there is a need to use methods that can account for multiple predictors and their interactions as well as limitations of the data available.

Machine learning (ML) techniques are powerful alternatives to conventional statistical regression models [10]. In ML, less restrictive algorithms are used to learn the relationship between outcome and proposed predictors [10]. Because of this flexibility, ML techniques are generally very effective in predicting outcomes at the individual level. Boosted regression trees (BRTs), a well-characterized ML technique, have been used in infectious disease modeling for data imputation [11]. The predictive performance of a BRT, its ability to identify influential predictors and interactions, and its ability to model nonlinear relationships make it an ideal technique to answer key questions in infectious disease epidemiology. For instance, Bhatt and colleagues [11] used BRTs to map the global distribution and burden of the dengue virus. Similarly, Dorigatti and colleagues [12] used BRTs to refine the efficacy estimate of the Sanofi Pasteur dengue vaccine CYD–TDV (chimeric yellow fever virus-DENV tetravalent dengue vaccine), illustrating the effectiveness of BRTs in estimating the uncertainty around missing or unrecorded data. However, despite these attempts, ML models are still seldomly used in the analysis and modeling of infectious diseases.

Here, we used BRTs to identify, using a nonparametric approach, the predictors of CFR for the West African epidemic, imputed unreported survival outcomes, and combined the imputed and reported survival outcomes to re-estimate CFRs for the epidemic. Finally, we compared the CFR estimates obtained without data imputation with estimates obtained including data imputation and estimates adjusted for model imperfection.

## METHODS

### Data Sources

We used case-level data that were reported to the WHO using the viral hemorrhage fever case-reporting forms as published in the supplementary data of the WHO Ebola Response Team [5]. On the case-reporting forms, cases were defined as confirmed, probable, and suspected using the WHO EVD case definition system [13].

Our main analyses present CFR inference based on confirmed, probable, and suspected cases, while sensitivity analyses using either “confirmed” cases or “confirmed and probable” cases are presented in the Supplementary Data (Supplementary Sensitivity Analysis). A wealth of information was recorded for each case, including demographic (eg, age, country) and clinical (eg, fever, diarrhea, bleeding) data.

The survival outcome for this dataset was reported as the “final status” of cases. Final status was defined as alive, dead, or missing. The fate of cases with missing final status was unknown (eg, cases could have been alive in the communities or later died in the communities but were not reported to, or recorded by, the appropriate authorities). Cases with known final outcomes accounted for 18 644 (56%) of the cases in the dataset.

We selected 43 variables as candidate predictors of CFR. This selection was informed by the literature [9, 14] as well as judgment on the influence of potential predictors on CFR.

### Statistical Methods

BRT models rely on decision trees and gradient boosting. With regression trees, a recursive binary split of data is carried out until a stopping criterion is reached [10]. Used alone for analysis, regression trees are susceptible to bias and inaccuracy. The gradient boosting applies randomness into the stage-wise fitting, which by introducing stochasticity helps to avoid overfitting of the model to the data (Supplementary Methods).

To fit the BRT model, we used the survival outcomes as the dependent variables and the candidate predictors as the independent variables. We evaluated the model performance in terms of multiple measures of predictive accuracy—that is, sensitivity, specificity, proportion of predictions correctly classified, and the area under the receiver operating characteristic curve (AUC). The performance measures quantify the ability of the BRT model to discriminate between outcomes (death/survival) for the given predictors [15]. For the AUC, performance values of 0.5 (50%) indicate random discrimination of outcomes and performance values of 1 (100%) indicate perfect discrimination of outcomes. These performance measures were used to determine the optimal hyperparameters.

**Figure 1.**
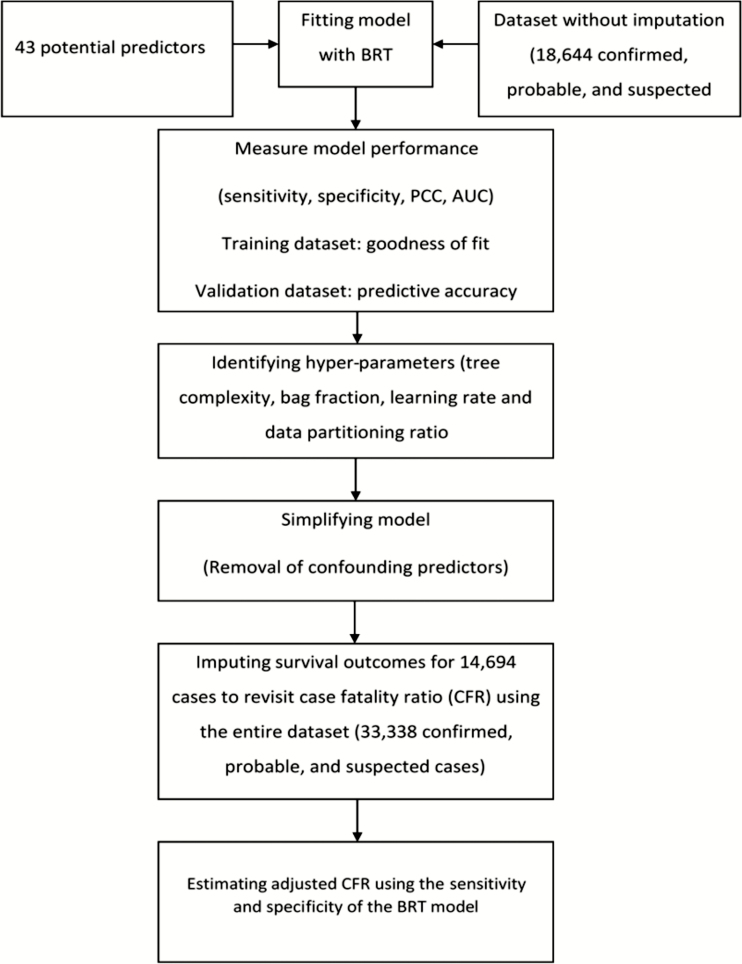
Schematic summary of the analysis steps used in this study. Abbreviations: AUC, area under the receiver operating characteristic curve; BRT, boosted regression tree; PCC, percentage correctly classified.

To identify the model hyperparameters (tree complexity [tc], learning rate [lr], and bag fraction [bf]), multiple BRT models were fitted using different parameter combinations. In total, we explored 72 hyperparameter combinations. Figure 1 presents a summary schematic of the analysis and further details of the parameterization are included in the Supplementary Data.

BRT models return probabilities for each outcome. Converting these probabilities into binary values requires a threshold probability [16]. We selected our threshold to obtain equal sensitivity and specificity. It allowed us make predictions without trading off death and survival outcomes.

We simplified the BRT model by excluding noninformative predictors with minimal effect on prediction [10]. The relative influence (RI) of a predictor is the number of times the predictor is selected to discriminate outcomes (death/survival) and the improvement in the model for each selection, which is based on the amount of residuals explained at each selection [17]. These RIs are scaled to sum to 100, and higher influences indicate greater effects on survival outcome. The model was repeatedly refitted, each time removing the predictor with the lowest RI. We applied a default rule that continued to remove predictors with the lowest RIs until the average change in predictive deviance exceeded the standard error of the full model (ie, model using all candidate predictors) [10].

The simplified model was used to predict the survival outcomes for the 44% of cases in the dataset with missing outcomes and the CFRs were re-estimated for the full dataset. Using nonparametric bootstrapping with replacement for 1000 realizations, we calculated the means and 95% confidence intervals (CIs) of the re-estimated CFRs (see Supplementary Methods).

CFR estimates with imputation ignore the bias arising from the imperfect sensitivity and specificity of the imputation algorithm. We therefore corrected our CFR estimate using the estimated sensitivity and specificity of the BRT model to obtain unbiased estimates [18]. The inferred CFR was calculated for each subset of interest according to the following formula:

$$\begin{array}{l} Inferred\;\;\;CFR=\frac{\left(q+specif\;icity-1\right)}{\left(sensitivity+specif\;icity-1\right)\;\;\;}, \end{array}$$

where q is the CFR for the imputed data (ie, data with unknown outcomes) using the estimated sensitivity and specificity of the BRT model [19, 20]. Thus, the final CFR was calculated after adjusting the CFR for missing outcomes for imputation sensitivity and specificity (Supplementary Methods).

## RESULTS

**Figure 2.**
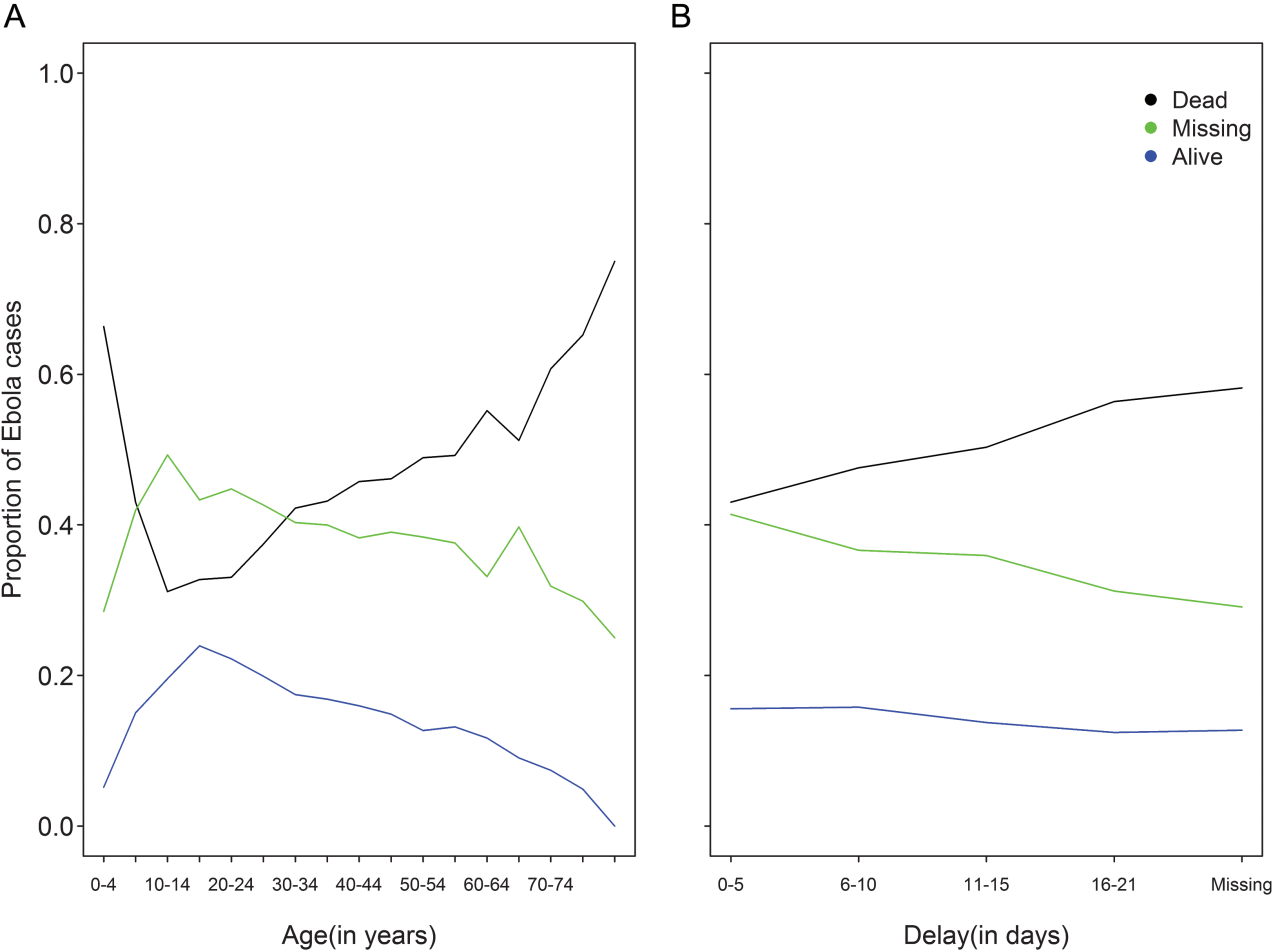
Proportion of known survival outcomes (ie, dead and alive) and unknown survival outcomes (ie, missing) for “confirmed, probable, and suspected” cases. *A*, Proportion of deaths, survivals, and entries with unknown outcome by age group (in years). *B*, Proportion of deaths, survivals, and entries with unknown outcome by reporting delay (in days).

In the dataset, a total of 18 644 (confirmed, probable, and suspected) cases with known outcome were reported out of 33 338 cases total (Table 1). The proportions of cases with dead, alive, and unknown survival outcomes vary across age classes and reporting-delay categories (Figure 2), with statistically significant differences indicating missingness departed from random (Supplementary Table 1).

**Table 1.** Case Fatality Ratio Estimates Without Imputation, Unadjusted With Imputation, and Adjusted With Imputation for Confirmed, Probable, and Suspected Cases

	Cases in the Dataset Without Imputation, n	Cases in the Dataset Including Those With Imputation, n	CFR Without Imputation, % (95% CI)	Unadjusted CFR With Imputation, % (95% CI)	Adjusted CFR With Imputation, % (95% CI)
Guinea	3740	3757	65.8 (61.6–69.9)	65.6 (61.3–69.6)	65.6 (61.3–69.6)
Liberia	4624	8130	71.7 (67.2–75.6)	69.7 (55.6–78.7)	79.2 (45.4–84.1)
Sierra Leone	10 280	21 451	81.3 (79.3–83.3)	74.6 (52.2–84.3)	89.1 (40.8–91.6)
Overall^a^	18 644	33 338	75.1 (73.5–76.6)	71.9 (56.1–79.8)	82.8 (45.6–85.6)

All CFR estimates and corresponding CIs were calculated using a nonparametric bootstrap of the BRT model.Abbreviations: BRT, boosted regression tree; CI, confidence interval; CFR, case fatality ratio.

^a^Adjusted CFR estimated using tp function from the R package RSurveillance to correct for bias in BRT model performance.

Using the 72 tested hyperparameter configurations of the full BRT model (ie, fitted using all available predictors), Supplementary Figures 1 and 2 show the out-of-sample predictive performance and the goodness-of-fit performance, respectively. Further analyses were based on the following optimal hyperparameters: data-partitioning ratio (*P* = .65), tree complexity (tc = 27), learning rate (lr = 0.001), and bag fraction (bf = 0.75). In the simplified model we retained 24 survival predictors (out of the 43 originally included in the full model), accounting for 96% of the RI of the full model (ie, model with all candidate predictors).

**Figure 3.**
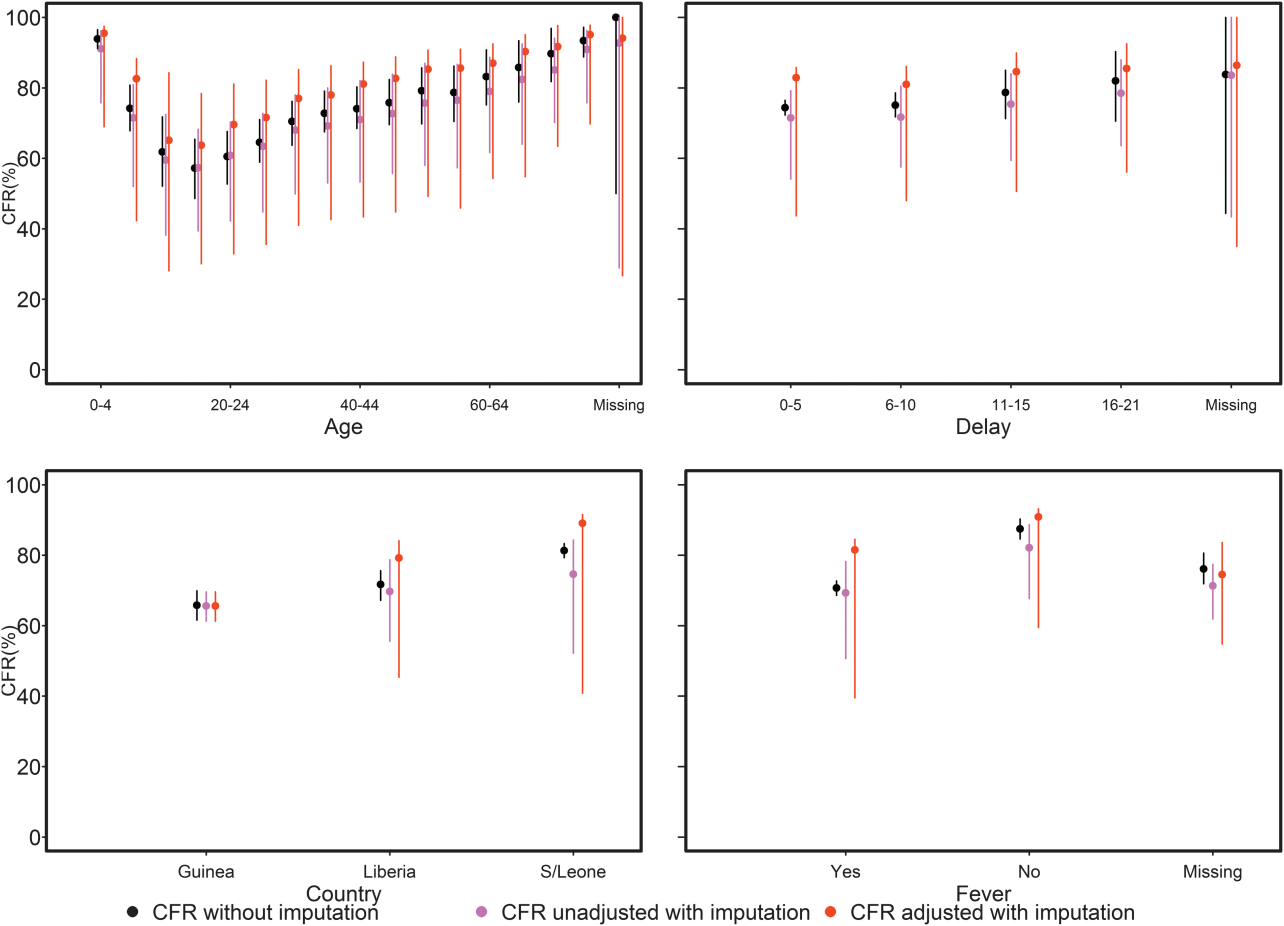
CFRs by age, delay, country, and fever. Median and 95% confidence intervals are plotted (based on 1000 bootstrap realizations for “confirmed, probable and suspected cases”). Abbreviations: CFR, case fatality ratio; S/Leone, Sierra Leone.

District, hospitalization status, age, case classification, and date of case reporting aggregated at three-month intervals (thereafter defined as quarter) were the most important predictors, with average RIs of 41.7%, 28.9%, 10.7%, 10.1%, and 2.2%, respectively (Table 2). Unadjusted CFR estimates (ie, CFR estimates obtained with imputation not corrected for imperfect sensitivity and specificity) and CFR estimates without imputation initially decreased with age, with the lowest CFR estimates found in the 15- to 19-year age group, with CFRs of 57.3% (95% CI, 39.4–68.2%) and 57.2% (95% CI, 48.6–65.4%) unadjusted and without imputation, respectively (Figure 3). Thereafter, CFR increased steadily with the highest rates in 75-years-and-older age group, with CFRs of 90.9% (95% CI, 75.8–96.3%) and 93.4% (95% CI, 88.8–97.2%) unadjusted and without imputation, respectively (Figure 3).

**Table 2.** Relative Contributions of the Predictors in the Minimal BRT Model Using 10-fold Cross-validation; tc = 27, lr = 0.001, and bf = 0.75; and Trained on 1000 Training Sets Generated by Randomly Sampling 65% of Cases With Known Survival Outcomes Without Bootstrapping

Predictors	Relative Contribution, %
District of origin	41.7
Hospitalization status	28.9
Age	10.7
Case Classification	10.1
Quarter	2.2
Delay	1.8
Anorexia	1.6
Difficult breathing	1.6
Fever	1.0
Fatigue	0.5

The minimal model used these 10 predictors.Abbreviations: bf, bag fraction; BRT, boosted regression tree; lr, learning rate; tc, tree complexity.

CFR estimates increased with increasing reporting delays. The lowest rates were at 0 to 5 days, with CFRs of 71.5% (95% CI, 54.1–79.2%) and 74.4% (95% CI, 72.3–76.5%) unadjusted and without imputation, respectively. The highest rates were at 16 to 21 days, with CFRs of 78.5% (95% CI, 63.6–88.0%) and 82.0% (95% CI, 70.6–90.3%) unadjusted and without imputation, respectively.

Figure 3 shows overall and country-level estimates. Overall CFR estimates were 71.9% (95% CI, 56.1–79.8%) and 75.1% (95% CI, 73.5–76.9%) unadjusted and without imputation, respectively (Table 1, Figure 3). Guinea had the lowest rates, with CFRs of 65.6% (95% CI, 61.3–69.6%) and 65.8% (95% CI, 61.6–69.9%) unadjusted and without imputation, respectively. The Liberian rates were lower, with CFRs of 69.7% (95% CI, 55.6–78.7%) and 71.7% (95% CI, 67.2–75.6%) unadjusted and without imputation, respectively. Sierra Leone had the highest rates, with CFRs of 74.6% (95% CI, 52.2–84.3%) and 81.3% (95% CI, 79.3–83.3%) unadjusted and without imputation, respectively (Table 1, Figure 3).

Fever, anorexia, difficult breathing, and fatigue are the clinical predictors in the minimal model (Table 2). The unadjusted CFR estimates were 69.3% (95% CI, 50.7–78.2%) and 82.1% (95% CI, 67.1–88.7%) for cases with fever and cases without fever, respectively (Figure 3). The CFR estimates without imputation were 70.7% (95% CI, 68.6–72.7%) and 87.5% (95% CI, 84.6–90.3%) for cases with fever and cases without fever, respectively (Figure 3). Fever, anorexia, difficult breathing, and fatigue together accounted for 4.7% of the RI of the minimal model, defined as the model using the 10 most influential predictors (district, hospitalization status, reporting delay, age, quarter, case classification, anorexia, difficult breathing, fatigue, and fever; see Table 2).

Adjusted CFRs (ie, corrected for imperfect sensitivity and specificity) with imputation for all predictors were broadly higher compared with unadjusted estimates and estimates without imputation (Figure 3). For instance, the adjusted CFR was 82.8% (95% CI, 45.6–85.6%) overall and they were 65.6% (95% CI, 61.3–69.6%), 79.2% (95% CI, 45.4–84.1%), and 89.1% (95% CI, 40.8–91.6%) for Guinea, Liberia, and Sierra Leone, respectively (Table 1).

In Table 3, we found that the full (ie, using 43 predictors), simplified (ie, using 24 predictors), and the minimal (ie, using 10 predictors) models produced comparable performances, for instance, the AUC of the full model was 76.7% (95% CI, 61.0–82.7%), that of the simplified model was 76.0% (95% CI, 56.8–82.1%), and that of the minimal model was 75.7% (95% CI, 56.1–82.1%).

**Table 3.** Boosted Regression Tree Model Performance

	Model Performance		
Performance Measures	Full Model^a^ With Bootstrap Median, % (95% CI)	Simplified Model^b^ With Bootstrap Median, % (95% CI)	Minimal Model^c^ With Bootstrap Median, % (95% CI)
Sensitivity	70.5 (56.0–76.5)	69.7 (52.5–75.6)	69.7 (51.7–75.7)
Specificity	70.5 (56.8–75.9)	69.8 (54.1–75.6)	69.8 (51.2–75.6)
PCC	70.5 (56.6–75.9)	69.9 (53.7–75.5)	69.7 (51.0–75.4)
AUC	76.7 (61.0–82.7)	76.0 (56.8–82.1)	75.7 (56.1–82.1)

Medians and 95% CIs are reported, based on 1000 bootstrap realizations using confirmed, probable, and suspected cases.Abbreviations: AUC, area under the receiver operating characteristic curve; BRT, boosted regression tree; CI, confidence interval; PCC, percentage correctly classified.

^a^The full BRT model used all 43 candidate predictors.

^b^The simplified BRT model used the 24 predictors retained after model simplification.

^c^The minimal BRT model used the 10 most important predictors.

## DISCUSSION

We have used the BRT to impute for unreported outcomes and produced improved (ie, representative of all cases and adjusted for BRT imputation performance) CFR estimates for the 2013–2016 West African Ebola epidemic. To our knowledge, the dataset used in this study (33 338 cases) is the largest case-level data used to characterize the CFR of EVD.

The effects of the BRT’s hyperparameters on the survival outcome were consistent with those in the published literature [10], with AUCs greater than 70% and less than 90% indicating appreciable predictive performance [21]. The data to which we fitted the BRT model were large relative to typical epidemiological datasets in outbreak settings; however, our method proved robust to changes in sample sizes, with comparable mean imputation performances reported even after data downsampling (Supplementary Figure 3).

Model simplification suggested that district, hospitalization status, age, case classification, quarter, reporting delay, anorexia, difficult breathing, fever, and fatigue were the most important predictors retained in the model. Most bleeding predictors (except for unexplained bleeding) were removed, which corroborates the existing literature suggesting that external bleeding was not a prominent characteristic of the West African Ebola epidemic [22–24]. Consequently, the predictive value of bleeding predictors was limited compared with the other (eg, age, reporting delay, district, and fever) predictors of CFR. Simplification removed some clinical predictors, but we found that the significance of clinical predictors varied with sample size and the subset of data included in the analysis. For example, hiccups occurrence was excluded from the final (simplified) model in our analysis but was found to have a predictive value in an earlier study conducted in Sierra Leone [22], although larger sample sizes would be needed to validate the significance of those results [22]. Earlier analyses of the West African epidemic showed that fever was not a significant predictor of the CFR [5, 9], while, in contrast, our study identified fever as the ninth most important predictor of the CFR. Further investigations revealed that the occurrence of fever only became a significant predictor in analyses using the datasets compiled after 25 November 2014 (Supplementary Table 2). From this finding we cannot determine whether the emergence of fever occurrence as an important predictor of the CFR is related to changing clinical presentation of the EVD over time or was caused by changes in reporting practices over the course of the epidemic. The high CFR in patients without fever might also suggest immune evasion [25, 26]. Interestingly, survival outcomes were better for patients with anorexia (see Supplementary Results), perhaps because these patients were better medically attended.

The predictors retained in the simplified model were broadly similar to those reported in the literature and the identified clinical predictors reflected the symptomatic profile of EVD cases during the West African epidemic and in previous outbreaks [9, 22, 27]. Demographic predictors of age, case classification, reporting delay, quarter, and district were important predictors of CFR, which broadly agrees with discussions in the literature suggesting that, notwithstanding the clinical presentations of EVD, demographic factors were predictors of EVD fatalities [9, 28, 29]. Age-dependent CFR variation, consistent with previous studies [6, 7], suggests a biological effect. Potential biological reasons include variation in the immunocompetency of children and young adults and the increased likelihood of comorbidities in aged individuals [25, 30]. The CFR increased with increasing reporting delay, suggesting that early case reporting could have improved survival outcomes, although previous findings have suggested that early reporting was insufficient to increase survival probability [7]. A clinical consideration is that people who were reported late may have been more likely to die because their symptoms persisted while those with milder symptoms would have recovered earlier and were less likely to report as patients with Ebola.

While adjusted CFRs were broadly higher than uncorrected CFRs, they corroborate with estimates of the Ebola *Zaire* CFR, from 1979 to 2014, compiled by Van Kerkhove and colleagues [1]. However, adjusted CFRs must be interpreted with appropriate caution, as CIs are wide. Results validating the sensitivity and specificity adjustments are reported in the Supplementary Data.

Country-level CFR estimates were consistent for Guinea, which had the highest proportion of complete outcome data (99.5%; ie, 3740 out of 3757 cases; see Table 1) among the 3 countries. The relatively high adjusted CFR estimates obtained in Liberia and Sierra Leone compared with estimates without and with imputation, and the associated relatively wide CI, could reflect the true CFR in these 2 countries but also possibly the changing data-collection practices over time. For instance, Liberia switched from an aggregate system of reporting to an individual-based system of reporting that could have affected data curation at the country level [31]. Potential biases may arise because definitive outcomes are more frequently reported for hospitalized cases, who, this study shows, were less likely to die from EVD infection [2]. Adjusted CFRs account for the estimated sensitivity and specificity of the BRT imputation model and the dependence of missingness on characteristics of the cases (eg, age and reporting delay).

These robust CFR estimates in conjunction with other resources can be used to inform public health contingency planning for future Ebola epidemics—for instance, to allocate resources and evaluate the effectiveness of interventions [32]. In epidemic settings with limited resources, as was the case in West Africa, reliable CFR estimates are essential for planning hospital care and managing cases [33].

While previous studies show clinical characteristics indicative of death with the intention of having a profile that could be used in subsequent case triaging [34, 35], the model built in this study cannot be used in such manner as it lacked clinically significant predictors, such as viral load, which were not captured in the dataset. Rather, the output of our analyses should be used to inform resource allocation and care planning. Also, our analysis provides a baseline CFR against which the CFR observed with interventions (such as a new treatment) could be assessed. As a known predictor of EVD CFR [6, 36, 37], viral load data, where available, should be used in future attempts to further refine CFR estimates of EVD. If some survival outcomes were incorrectly recorded during data collection and/or if some cases were missing entirely from the database, then, in the absence of at least some gold-standard data, no estimation strategy can fully account for such limitations and this may be in part responsible for the imperfect nature of the imputation process.

In conclusion, we have demonstrated that BRT modeling can be used to obtain improved estimates of CFR where outcome data are unreported. This BRT modeling framework could be implemented to further investigate EVD CFR heterogeneity and adapted to other infectious disease epidemiological analyses based on datasets with missing data.

## Supplementary Data

Supplementary materials are available at *Clinical Infectious Diseases* online. Consisting of data provided by the authors to benefit the reader, the posted materials are not copyedited and are the sole responsibility of the authors, so questions or comments should be addressed to the corresponding author.

ciz678_suppl_Supplementary_Figure_1

ciz678_suppl_Supplementary_Figure_2

ciz678_suppl_Supplementary_Figure_3

ciz678_suppl_Supplementary_Figure_Legends

ciz678_suppl_Supplementary_Material

## Notes

***Author contributions.*** A. F., P. N., I. D., and C. A. D. conceived and designed the experiments and wrote the paper. A. F. performed the experiments and analyzed the data.

***Acknowledgments*.** The study was based on data routinely collected by national and international staff in conjunction with World Health Organization (WHO). The authors thank all those involved in collecting, entering, and cleaning the data, including members of the WHO Ebola Response Team.

***Financial support.*** This work was supported by the Commonwealth Scholarship Commission, the UK Medical Research Council and the Department for International Development (center funding) (grant number MR/R015600/1), the National Institute of Health Research, and an Imperial College Junior Research Fellowship.

***Potential conflicts of interest.*** The authors report no potential conflicts of interest. All authors have submitted the ICMJE Form for Disclosure of Potential Conflicts of Interest. Conflicts that the editors consider relevant to the content of the manuscript have been disclosed.
