# Supplementary figures and images for: Case Fatality Ratio Estimates for the 2013–2016 West African Ebola Epidemic: Application of Boosted Regression Trees for Imputation
Source: Clin Infect Dis. 2019 Jul 22;70(12):2476–83. doi: 10.1093/cid/ciz678 (PMC7286386; doi:10.1093/cid/ciz678)

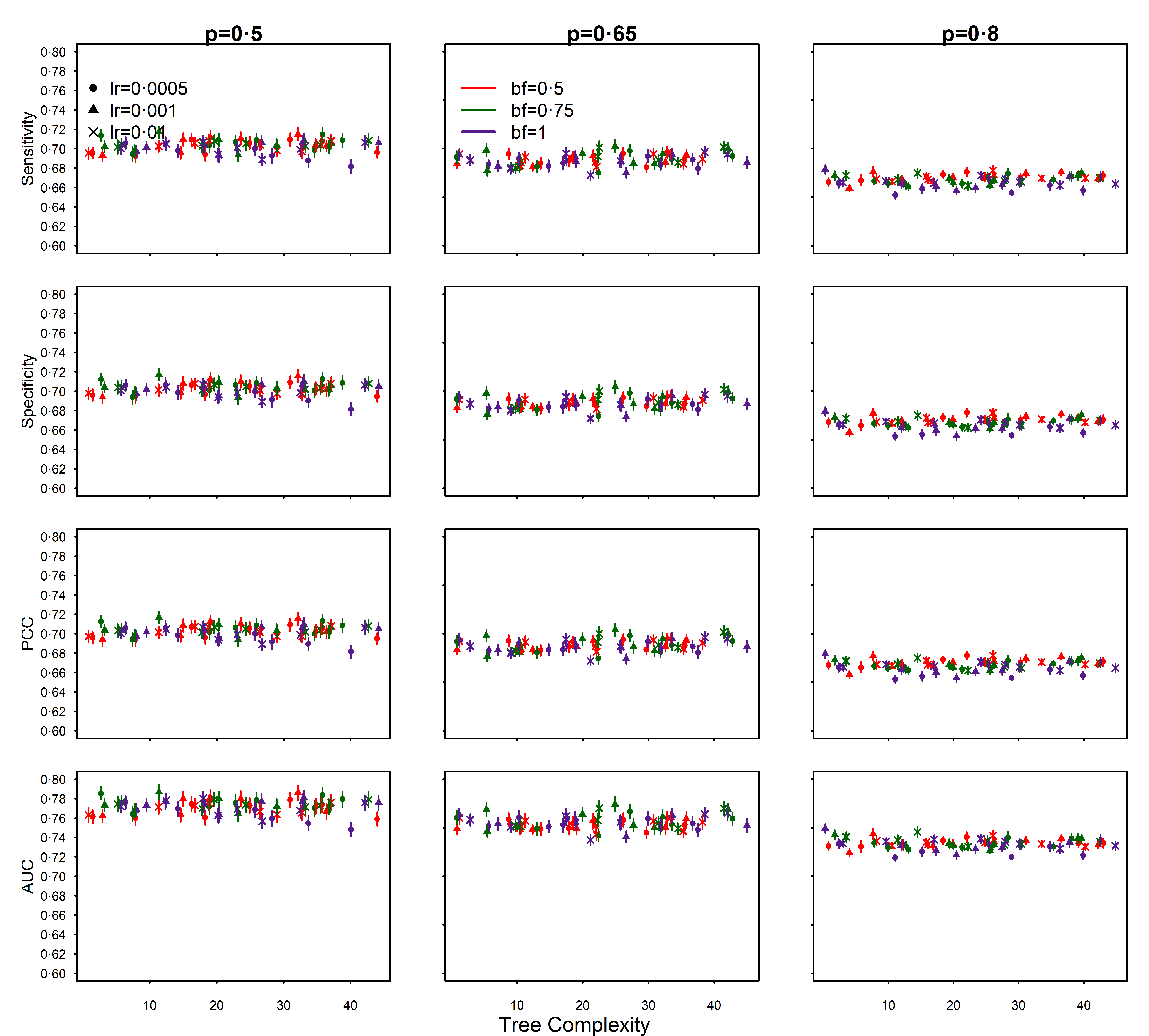

Supplement: ciz678_suppl_Supplementary_Figure_1 [file ciz678_suppl_supplementary_figure_1.png]

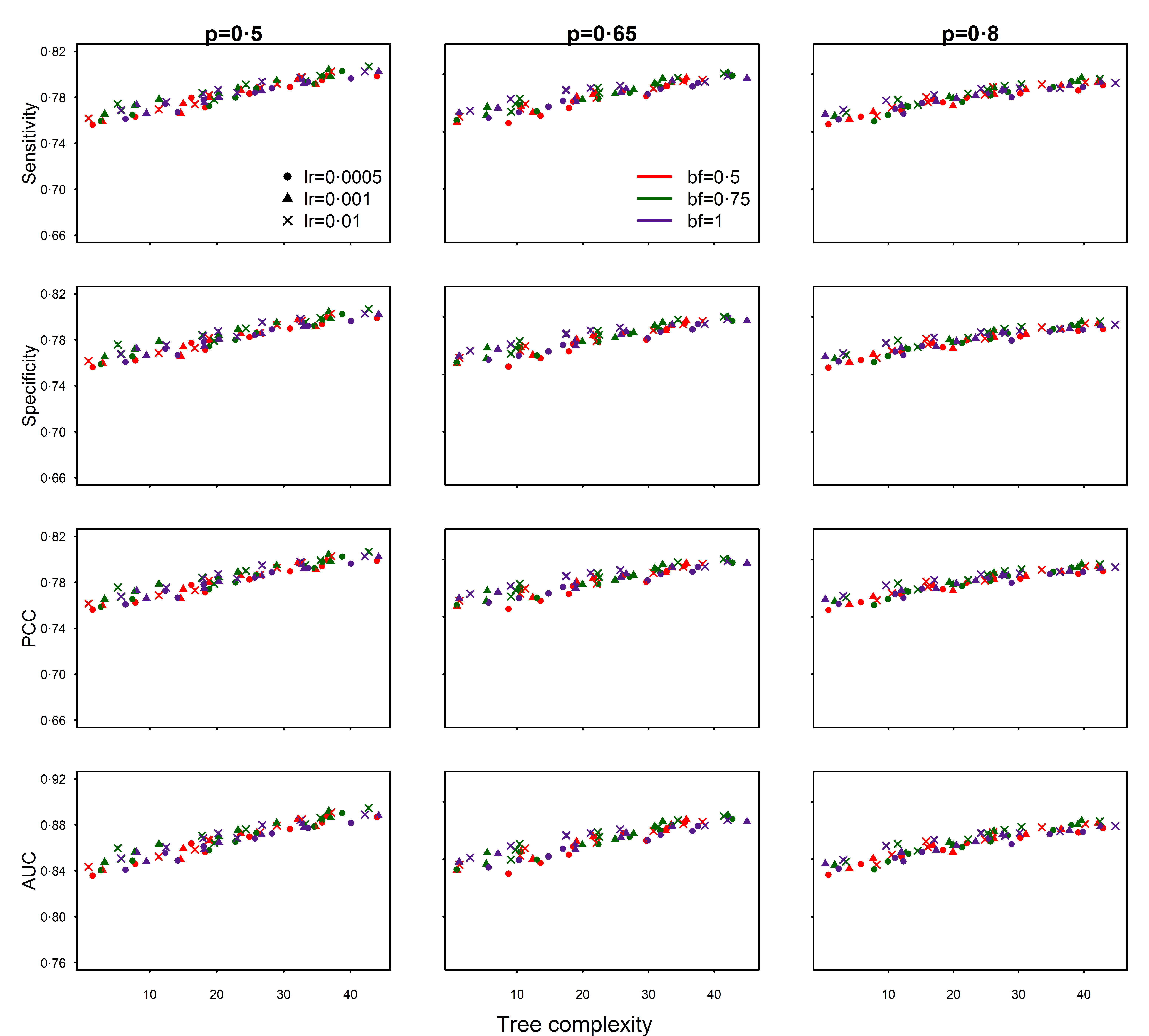

Supplement: ciz678_suppl_Supplementary_Figure_2 [file ciz678_suppl_supplementary_figure_2.png]

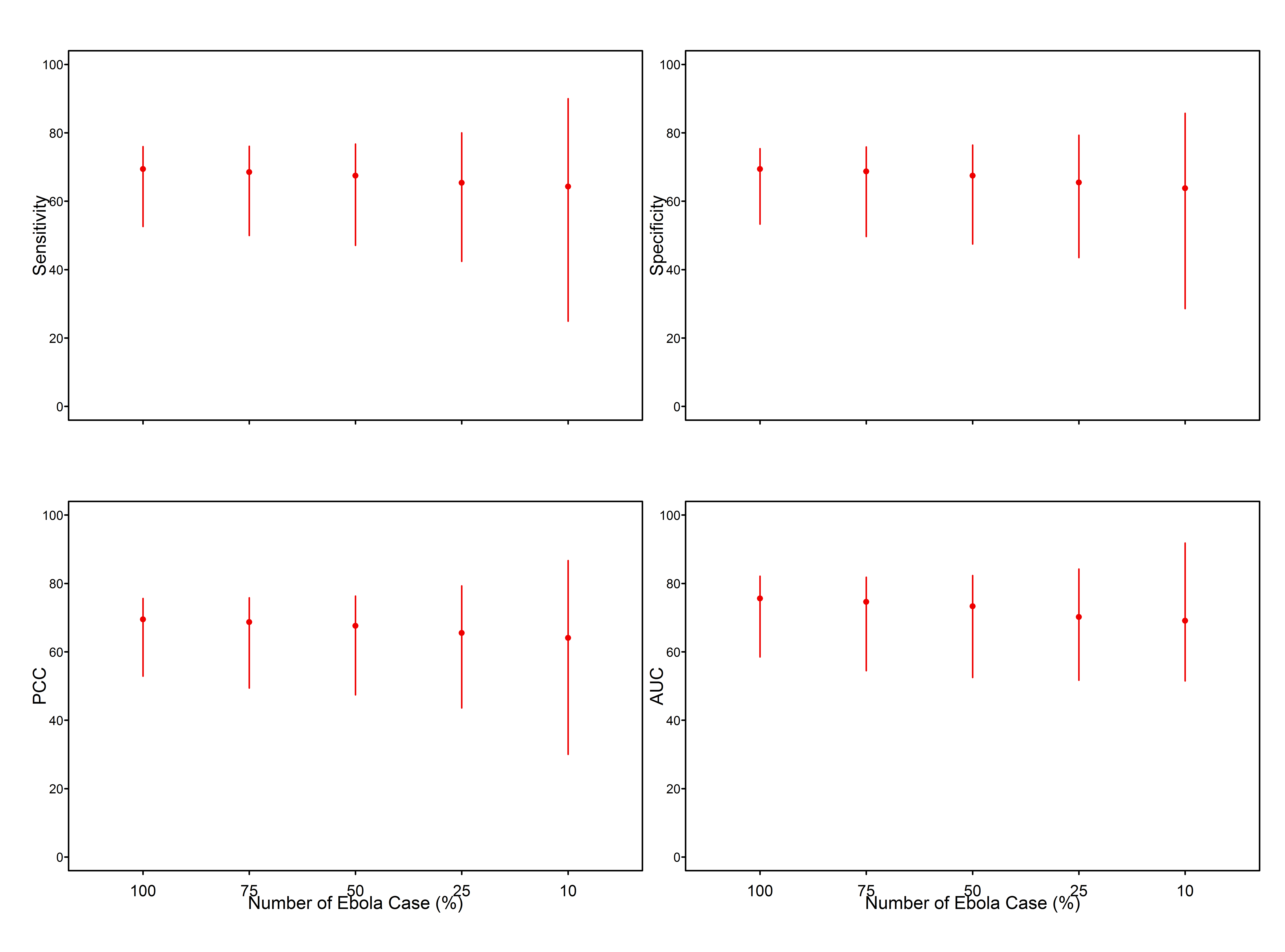

Supplement: ciz678_suppl_Supplementary_Figure_3 [file ciz678_suppl_supplementary_figure_3.png]
