# Supplementary material for: Case Fatality Ratio Estimates for the 2013–2016 West African Ebola Epidemic: Application of Boosted Regression Trees for Imputation
Source: Clin Infect Dis. 2019 Jul 22;70(12):2476–83. doi: 10.1093/cid/ciz678 (PMC7286386; doi:10.1093/cid/ciz678)
Supplement: ciz678_suppl_Supplementary_Figure_Legends [file ciz678_suppl_supplementary_figure_legends.docx]

**Supplementary Figure 1: Out-of-sample predictive accuracy measures (sensitivity, specificity, proportion of predictions correctly classified (PCC) and area under the receiver operating curve (AUC)) on the validation data obtained using 72 different hyperparameterisations of the BRT model. *p* represents the proportion of data to train the model with *p*=0.5*, p*=0.65 and *p*=0.8 explored.**

**Supplementary Figure 2: Goodness-of-fit accuracy measures (sensitivity, specificity, proportion of predictions correctly classified (PCC) and area under the receiver operating curve (AUC)) on the training data obtained using 72 different hyperparameterisations of the BRT model. p represents the proportion of data to train the model with p=0.5, p=0.65 and p=0.8 explored.**

**Supplementary Figure 3:** **Performance (i.e. sensitivity, specificity, PCC, and AUC) of BRT model after down-sampling of data for all Ebola cases (i.e. confirmed, probable and suspected).**
