# Supplementary material for: Case Fatality Ratio Estimates for the 2013–2016 West African Ebola Epidemic: Application of Boosted Regression Trees for Imputation
Source: Clin Infect Dis. 2019 Jul 22;70(12):2476–83. doi: 10.1093/cid/ciz678 (PMC7286386; doi:10.1093/cid/ciz678)
Supplement: ciz678_suppl_Supplementary_Material [file ciz678_suppl_supplementary_material.docx]

**Supplementary Data**

**Case fatality ratio estimates for the 2013 – 2016 West African Ebola epidemic: application of Boosted Regression Trees for imputation**

**Authors**: Alpha Forna^1^, MSc, Pierre Nouvellet^1,2^, PhD, Ilaria Dorigatti^1*^, PhD, and Christl A. Donnelly^1,3*^, ScD

**Affiliations: 1.** MRC Centre for Global Infectious Disease Analysis, Department of Infectious Disease Epidemiology, Imperial College London, London, United Kingdom.

**2.** School of Life Sciences, University of Sussex.

**3.** Department of Statistics, University of Oxford.

^*^These authors contributed equally.

**Correspondence to:** Alpha Forna

**Full Address**: MRC Centre for Global Infectious Disease Analysis, Department of Infectious Disease Epidemiology, Imperial College London, London, United Kingdom**.**

**Email:** [a.forna16@imperial.ac.uk](mailto:a.forna16@imperial.ac.uk)

**Alternate corresponding author:** Christl A. Donnelly

**Full Address**: Department of Statistics, University of Oxford, Oxford, UK and MRC Centre for Global Infectious Disease Analysis, Department of Infectious Disease Epidemiology, Imperial College London, London, United Kingdom.

**Email:** [christl.donnelly@stats.ox.ac.uk](mailto:christl.donnelly@stats.ox.ac.uk) and c.donnelly@imperial.ac.uk

# Table of contents

[Table of contents 2](#_Toc11834796)

[1 Details of Methods 3](#_Toc11834797)

[1.1 Case definition for Ebola virus disease (EVD) cases 3](#_Toc11834798)

[1.2 Conceptual description of BRT 3](#_Toc11834799)

[1.3 K-fold cross validation 3](#_Toc11834800)

[1.4 Summary description of BRT parameters used to optimize performance 3](#_Toc11834801)

[1.5 Non**-**parametric bootstrapping 4](#_Toc11834802)

[1.6 Algorithm implemented for hyperparameter tuning 5](#_Toc11834803)

[1.7 Algorithm implemented for CFR with imputation estimation 5](#_Toc11834804)

[1.8 Algorithm implemented for CFR adjusted for BRT model bias 6](#_Toc11834805)

[1.9 Algorithm implemented to validate sensitivity/specificity adjustment 6](#_Toc11834806)

[2 Additional results 8](#_Toc11834807)

[2.1 Comparison of age and reporting-delay between cases with known and unknown outcome. 8](#_Toc11834808)

[2.2 CFR by fever occurrence and date of case reporting 9](#_Toc11834809)

[2.3 CFR without imputation for individual predictors 10](#_Toc11834810)

[2.4 Proportion of predictors with known and unknown survival outcomes 11](#_Toc11834811)

[2.5 Identification of hyperparameters. 15](#_Toc11834812)

[2.6 Robustness of BRT imputation to data down sampling 16](#_Toc11834813)

[2.7 Comparison of CFR estimates obtained on the training set, without and with imputation 17](#_Toc11834814)

[2.7.1 Confirmed, probable, and suspected cases 18](#_Toc11834815)

[2.7.2 Confirmed and probable cases. 20](#_Toc11834816)

[2.7.3 Confirmed cases. 22](#_Toc11834817)

[2.8 Comparison of CFR estimates obtained on the validation data, with and without imputation 24](#_Toc11834818)

[2.8.1 Confirmed, probable, and suspected cases 24](#_Toc11834819)

[2.8.2 Confirmed and probable cases 26](#_Toc11834820)

[2.8.3 Confirmed cases 28](#_Toc11834821)

[2.9 CFR adjusted with imputation by gender and age (categories) 30](#_Toc11834822)

[2.10 CFR estimates without, unadjusted and adjusted with imputation for other predictors in simplified model 30](#_Toc11834823)

[2.8.1 Confirmed, probable and suspected cases 31](#_Toc11834824)

[2.8.2 Confirmed and probable cases 32](#_Toc11834825)

[2.8.3 Confirmed cases 33](#_Toc11834826)

[2.11 Sampling to explore simulated missingness 34](#_Toc11834827)

[3 Sensitivity Analysis 36](#_Toc11834828)

[3.1 Comparison of known and unknown outcome data for ‘confirmed’ and ‘confirmed and probable’ cases 36](#_Toc11834829)

[3.2 BRT model parameterisation for confirmed case data 36](#_Toc11834830)

[3.3 Minimal model predictors for variation in case definition 39](#_Toc11834831)

[3.4 CFRs for variations in case definition 39](#_Toc11834832)

[3.5 BRT imputation performance for variations in case definition 40](#_Toc11834833)

[3.6 CFR estimates by age group, delay, country and fever for other case definitions. 41](#_Toc11834834)

[3.6.1 Confirmed and probable cases 41](#_Toc11834835)

[3.6.2 Confirmed cases 42](#_Toc11834836)

[4 References 42](#_Toc11834837)

# 1 Details of Methods

1.1 Case definition for Ebola virus disease (EVD) cases

Confirmed Cases: Confirmed cases are defined as persons who returned a positive laboratory test for EVD [1].

Probable Cases: Probable cases are defined as persons suspected to have EVD as evaluated by a clinician or persons who died from suspected Ebola and had epidemiologic link to confirmed cases. Such persons need not to have been tested or confirmed by laboratory [1].

Suspected Cases: Suspected cases are defined as persons, alive or dead, with sudden onset of high fever and had contact with suspected, probable, or confirmed EVD cases or with a dead or sick animal; persons with sudden onset of high fever and a minimum of three of the following symptoms: headache, vomiting, anorexia or loss of appetite, diarrhoea, lethargy, stomach pain, aching muscles or joints, difficulty swallowing, breathing difficulties, or hiccupping; or any person who had unexplained bleeding or who died suddenly from an unexplained cause [1].

## 1.2 Conceptual description of BRT

BRTs combine regression/classification trees with “boosting”. For regression trees, a set of decision rules are built on the predictors. These decision rules are constructed by recursively splitting the data into successive smaller subgroups using binary splits. For all predictors, splits are repeatedly applied to output until the best splits are identified. The best splits are those that maximise the homogeneity of the smaller subgroups with respect to the outcome (e.g. death/survival). For the classification trees used in this study, the splits are the tree branches and the homogeneous subgroups are terminal nodes “leaves” of the tree. The boosting part of the model is a form of gradient descent that adds new trees to reduce the gradient of the loss function– a measure indicating the loss in imputation performance due to a suboptimal model, thus further reducing variance in the outcomes. The final model is an additive model in which each term is a tree [2]. Because this is a forward stage-wise fitting process, cross validation (more details provided in section 1.3) can be applied to further optimise the number of trees and prevent overfitting. The BRT algorithm implemented in the “gbm package” used in this study can be found here [3]. Briefly, this algorithm depends on four hyperparameters (tree-complexity, data partitioning ratio, learning rate and bag fraction), changing these hyperparameters affects the model performance. A short description of each hyperparameter is given below, with more details provided in section 1.4. Tree complexity determines the number of nodes in each tree. The data partitioning ratio determines the proportion of data used for model training and out-of-sample validation. The learning rate is a scaling factor used to scale the predictions of each model. The smaller the learning rate, the less importance given to each single tree in the model. Bag fraction introduces stochasticity to the model thus preventing overfitting. To optimise model performance, the tree complexity, data partitioning ratio, learning rate and bag fraction were all varied.

## 1.3 K-fold cross validation

K-fold cross validation is the way overfitting is monitored and avoided when fitting BRT models. Here we implemented a 10-fold cross validation, which means that the training data were divided into 10 subsets and at each fold the model was trained on 9 (out of the 10) unique subsets and the omitted subset was used for hold-out testing [4]. The average prediction error across the testing subsets is a measure of goodness of fit. This allows us to identify overfitting and determine the performance of model parameters.

## 1.4 Summary description of BRT parameters used to optimize performance

***Tree Complexity (tc)***

The tree complexity (tc) sets the number of nodes in each tree of the model. tc= 1 implies that each tree contains one node and two leaves and tc=2 implies that the tree contains 2 nodes and 4 leaves. The optimal number of trees is a function of both the learning rate and tree complexity [4]. In the grid search used for optimal hyperparameterisation we included tc between 5 to 40 at intervals of 5. Furthermore, the tc defines the interactions in the BRT model, for a tc=1, each tree contains only one stump (predictor) and so a collection of trees will behave as just an additive model of individual predictors. However, for tc>1 more predictors are used for each tree and each tree could be regarded as an interaction term of predictors. Interactions are inherently included when building BRT models and the probabilities at each terminal node reflects these interactions in the model.

***Learning Rate (lr)***

The learning rate (lr), also called shrinkage factor, determines the contribution of each tree to the final model [4]. The smaller the learning rate, the less each individual tree influences the final model and therefore, a larger number of trees is generally needed to minimise the hold-out deviance and a larger computation time is needed. In the grid search used for optimal hyperparameterisation we included lr of 0.01, 0.001 and 0.0005, as suggested in the literature [3].

***Bag fraction (bf)***

Bag fraction (bf) introduces stochasticity into a BRT model which improves accuracy and reduces overfitting. Reducing overfitting introduces variance to the fitted values [4]. With a default of 0.5, 50% of observations are selected without replacement at each iteration. When the bf=1, no stochasticity is introduced. Optimal bf (0.5 to 0.75) has been suggested in the literature [4]. In the grid search used for optimal hyperparameterisation we included bf between 0.5 to 1 at intervals of 0.25.

***Data Partitioning Ratio***

In this analysis, we tested three data partitioning approaches to select the optimal hyperparameterisation. These were as follows:

**Pareto’s Principle (80/20 partitioning)**

We used 80% of the data for training the model and remaining 20% for out-of-sample validation.

**Freidman’s (65/35 or 70/30** partitioning)

We used 65% of the data for training the model and remaining 35% for out-of-sample validation [5]. A slight variant of this is the 70% training and 30% validation [6]. In our grid search we used the 65/35 partitioning.

**Half and Half Partitioning (50/50 partitioning)**

We used 50% of the data for training the model and remaining 50% for out-of-sample validation.

We varied tree complexities from 5 to 40 in steps of 5, learning rates of 0.01, 0.001 and 0.0005 and bag fractions from 0.5 to 1 in steps of 0.25. Additionally, to optimise the data partitioning for model training and validation, we explored three partitioning ratios (p) (0.5, 0.65, and 0.8).

## 1.5 Non**-**parametric bootstrapping

The bootstrap is a widely applicable and very powerful statistical tool to estimate the uncertainty around estimates. Non-parametric bootstrapping allows us to calculate uncertainty using the original dataset without predefining the underlying distribution function. For an original dataset of $n$ cases, we randomly sample $n$ cases with replacement to produce a bootstrap dataset. This random sampling is repeated $B$ (e.g. 1000 times) times and the final mean/median estimates and corresponding confidence interval are calculated from these $B$ bootstrap datasets [7]. In the context of this study, we applied the non-parametric bootstrap to our modelling framework, generated 1000 CFR estimates with imputation and from these calculated the median and 95% confidence interval.

Within our modelling framework, we divided the data into, data with known survival outcomes and data with unknown survival outcomes. The data with known survival outcomes were further divided into training and out-of-sample datasets. We fitted the model on the training data and measured out-of-sample performance on the out-of-sample data. Relying on this out-of-sample performance, we used the BRT model to impute for the data with unknown outcomes. CFR estimates were then calculated combining both the imputed and known survival outcomes. Bootstrapping allowed us to quantify the variance of these CFR estimates, as we repeatedly (1000 realizations) sampled all the data with replacement before we divided the data into the initial two parts (i.e. data with known and data with unknown outcomes), fitted the model and imputed for the unknown outcomes. In bootstrapping, because we sampled with replacement each time, cases in the training data could have appeared in the out-of-sample data thus producing over optimistic out-of-sample performance and unreliable CFR estimates that are not a true reflection of the imputation potential of the model. In our implementation, by ensuring that for each bootstrap dataset, cases in training data were excluded from the out-of-sample dataset, the out-of-sample performance and corresponding robust CFR estimates we estimated are a true reflection of the imputation potential of the BRT model.

## 1.6 Algorithm implemented for hyperparameter tuning

For given values of $tc$, $lr$ and $bf$, and the proportions ($p$) of cases in training set, BRT models were built and validated using the following algorithm:

1. Generate the training set by randomly sampling without replacement the specified proportions of cases with death and survival outcomes.
2. Generate the out-of-sample validation set, consisting of the cases with observed survival outcomes excluded from the training set (step 1).
3. Build the BRT model through the gbm.step function in the ‘dismo’ package, using the training set built in step 1, the specified $tc$, $lr$, $bf$, 10-fold cross-validation and increasing the model in steps of 50 trees at each iteration, using survival outcome (i.e. death or survival) as response variable and gender, health care worker, health care worker position, bleeding gums, bleeding nose, bleeding skin, bleeding stool, bleeding urine, haematemesis, vomiting, cough, bleeding from other sites, bleeding injection site, bleeding vagina, unexplained bleeding, fever, jaundice, painful eyes, rash, unconsciousness, fatigue, anorexia, vomiting, diarrhoea, headache, abdominal pain, muscle pain, joint pain, chest pain, coughing blood, difficult breathing, conjunctivitis, sore throat, confused, hiccup, difficulty swallowing, case classification, quarter, age, delay, current hospitalisation, districts and country of origin as predictors. The optimal number of trees minimising the holdout (cross-validation) deviance is computed.
4. Use the BRT model built in step 3 to predict the survival outcomes of the cases in the validation set. For each subject $i$ in the validation set, the BRT model gives the probability $p$ that subject $i$is dead.
5. Define the cut-off threshold $p_{T}$ , by which cases with $p_{i}$ >$p_{T}$ are classified as dead or alive otherwise. We chose cut-off values giving equal sensitivity and specificity using the optimal.threshold function in the PresenceAbsence package.
6. Use the cut-off threshold $p_{T}$ computed in step 5 to classify cases in the validation set as dead or alive.
7. Compute the sensitivity (proportion of deaths correctly classified), specificity (proportion of survivals correctly classified) and percentage of predictions correctly classified in the validation set PCC and the area under the receiver operating characteristic curve (AUC).

The choice of optimising the cut-off thresholds to have equal sensitivity and specificity in step 5 was made to avoid introducing bias in CFR estimated with imputation.

## 1.7 Algorithm implemented for CFR with imputation estimation

Mean CFR estimates with imputation

The mean CFR estimates with imputation were calculated from 1,000 realisations of steps 1 – 7 (appendix p 6), followed by steps 8, 9 and 10 below:

1. Use the BRT model built in step 4 to impute for cases with missing survival outcomes.
2. Use the cut-off threshold $p_{T}$ computed in step 6 to classify imputed cases as dead or alive.
3. Calculate the CFR using the cases with both imputed and observed survival outcomes.

Confidence interval

Confidence intervals around the mean CFR estimates were calculated by bootstrapping. Using the optimal tuning parameter set ($tc$ = 27, $lr$ = 0.001, $bf$ = 0.75), the 95% CI of the CFR estimates with imputation were calculated as the 2.5-97.5 percentiles of 1,000 random realisations of the algorithm detailed below (steps 2 – 9 below are the same as steps 1 – 7 (appendix p 6):

1. Generate a synthetic population by sampling with replacement the 18,644 confirmed, probable, and suspected cases in the Ebola epidemic line list (confirmed and confirmed and probable for sensitivity analysis).
2. Generate the training set by sampling without replacement 65% of cases from the synthetic population sampled in step 1 with known survival outcomes.
3. Generate the out-of-sample validation set – i.e. cases in the synthetic population generated in step 1 with known survival outcomes who are not included in the training set (step 2).
4. Build the BRT model through the gbm.step function in the dismo package, using the training set build in step 3, $tc$ = 27, $lr$= 0.001, $bf$= 0.75, 10-fold cross-validation and increasing the model in steps of 50 trees at each iteration, using survival outcome as response variable and using the predictors in the simplified model as predictors.
5. Use the BRT model built in step 4 to predict survival outcomes in the validation set. For each subject $i$in the validation set, the BRT model calculates the probability $p$ that subject $i$is dead.
6. Define the cut-off threshold $p_{T}$ , by which cases with $p_{i}$ > $p_{T}$ are classified as dead or alive otherwise. We chose cut-off values giving equal sensitivity and specificity using the optimal.threshold function in the PresenceAbsence package.
7. Use the cut-off thresholds$p_{T}$ computed in step 6 to classify the cases in the validation set generated in step 3 as dead or alive.
8. Compute the sensitivity (proportion of dead cases correctly classified), specificity (proportion of case that survived correctly classified) and percentage of predictions correctly classified in the validation set and the area under the receiver operating characteristic curve (AUC).
9. Use the BRT model built in step 4 to impute the survival outcomes of the cases in the synthetic prediction set (i.e. the cases sampled in step 1 with missing survival outcomes).
10. Calculate the CFR among the cases with complete and imputed survival outcomes.

## 1.8 Algorithm implemented for CFR adjusted for BRT model bias

Mean Adjusted CFR estimated

The mean CFR estimates adjusted for bias in BRT model were calculated from 1,000 realisations of steps 1 – 7 (appendix p 6), followed by steps 8,9,10 and 11 below:

1. Generate cases with just the imputed death outcomes.
2. Use the dead cases in step 8, the total number of cases in the imputed data and tp function in the RSurveillance package to estimate the inferred CFR.
3. Multiply the number of cases in the imputed data by the inferred CFR to get the number of inferred deaths $f_{T}$.
4. Adjusted CFR is the ratio of the sum of $f_{T}$ and the number of deaths in data with known survival outcomes, divided by the sum of all deaths and survivals in the complete data (i.e. data with both imputed and observed outcomes).

Confidence interval

Although the tp function of the “RSurveillance” package provides parametric approaches to estimate confidence intervals[8], in our implementation, confidence intervals around the mean adjusted CFR estimates were calculated by non-parametric bootstrapping (see section 1.5). Using the optimal tuning parameter set ($tc$ = 27, $lr$ = 0.001, $bf$= 0.75), the 95% CI of the adjusted CFR estimates were calculated as the 2.5-97.5 percentiles of 1,000 random realisations of the algorithm detailed in both steps 1 – 7 (appendix p 6) and 8 – 11 (appendix p 6).

## 1.9 Algorithm implemented to validate sensitivity/specificity adjustment

The mean CFR for simulated missingness were calculated from 1000 realisations of step 1-7 (appendix p 6), followed by steps 8 to 17 below:

8. Generate subset of data $d_{1}$containing only cases with observed deaths.

9. Generate a new variable for $d_{1}$from a uniform distribution with minimum 0 and maximum 1 and order $d_{1}$ by this new variable.

10. Sample from $d_{1}$ (without replacement) cases in the proportion of (inferred CFR) estimated after adjusting for sensitivity and specificity as described previously (appendix p 6).

11. Generate a subset of data $d_{2}$ containing only cases with observed survivals.

12. Generate a new variable for $d_{2}$ from a uniform distribution with minimum 0 and maximum 1 and order $d_{2}$ by this new variable

13. Sample from $d_{2}$ (without replacement) cases in the proportion of (1–inferred CFR) estimated after adjusting for sensitivity and specificity as described previously (appendix p 7).

14. Combine $d_{1}$and $d_{2}$to create a full data $d_{x}$ with simulated missingness.

15. Use the BRT model in step 1-7 in section 1.6 to predict survival probabilities for $d_{x}$.

16. Use optimal threshold $p_{T}$ from the BRT model to convert survival probabilities to binary alive and survival outcomes.

17. Estimate CFR of the full data $d_{x}$and compare with CFR of only the imputed outcomes for BRT model in step 1-7.

Confidence interval

The confidence interval around the mean CFR for simulated missingness was calculated by non-parametric bootstrapping(i.e. sample the data with replacement each time we run the algorithm). The 95% CI is calculated as the 2.5-97.5 percentiles of 1,000 random realisations of the algorithm.”

# 2 Additional results

## 2.1 Comparison of age and reporting-delay between cases with known and unknown outcome.

We used a two-sample t-test to compare cases with known and unknown survival outcomes in the same confirmation status categories (Table S1) and characterised the distribution of the data. This allowed us to investigate whether survival outcome missingness is independent of the characteristics of the cases. The results show that outcomes are not missing completely at random and that the missingness depends on the observed predictors. Thus, the use of BRT to impute outcomes (death and survival) was a valid approach.

Table S1: Summary statistics for known outcomes and unknown outcomes data for age and reporting-delay. Known outcomes data are data without imputation and unknown outcomes data are data for which we conducted imputation. Reporting-Delay: delay between date of reporting and the date of the onset of symptoms (in days). We used an independent two-sample t-test (p values) to compare known outcomes data and unknown outcomes data.

| Predictors | Confirmed Cases | | | Confirmed and Probable Cases | | | Confirmed, Probable and Suspected | | |
| --- | --- | --- | --- | --- | --- | --- | --- | --- | --- |
|  | Known Outcomes*  Median(IQR) | Unknown Outcomes ^a^ Median(IQR) | p-value | Known Outcomes*  Median(IQR) | Unknown Outcomes ^a^ Median(IQR) | p-value | Known Outcomes*  Median(IQR) | Unknown Outcomes ^a^ Median(IQR) | p-value |
| Age (in years) | 30.0 (19.0-44.0) | 28 (17.0-40.0) | <0.0001 | 30.0 (19.0-45.0) | 28.0 (16.0-40.0) | <0.0001 | 30.0 (14.0-46.0) | 29.0 (17.0-43.0) | <0.0001 |
| Reporting-Delay (in days) | 4.0 (2.0-6.0) | 3.0 (2.0-5.0) | <0.0001 | 4.0 (2.0-6.0) | 3.0 (2.0-6.0) | <0.0001 | 4.0 (2.0-7.0) | 3.0 (2.0-6.0) | <0.0001 |

## 2.2 CFR by fever occurrence and date of case reporting

The earliest and latest dates of case reporting in the dataset are 2^nd^ January 2014 and 25^th^ September 2015, respectively. Informed by the latest date (25^th^ November 2014) from the analyses in–West African Ebola Epidemic After One Year: Slowing But Not Yet Under Control [1, 9], and starting from the earliest date 2^nd^ January 2014, we used a cut-off date of 25^th^ November 2014 to estimate CFR for before and after the cut-off. Table S2 shows the CFR estimates obtained in the time windows 2^nd^ January to 25^th^ November 2014 and 26^th^ November 2014 to 25^th^ September 2015. The CFR estimates by fever occurrence in the time window 2^nd^ January 2014 to 25^th^ November 2014 shows that the estimates obtained in this study are consistent with the estimates obtained in previous publications [1, 9]. Furthermore, these results show that both before and after the cut-off date CFR was higher in patients with no occurrence of fever. Perhaps, this is due to fever not being measured in patients found dead in the community or arriving dead at the ETC.

Table S2: CFR by fever occurrence and date of reporting. The cut-off date (25^th^ November 2014) was informed by previous work using the same data but for an earlier stage of the epidemic.

| Case Definition | CFR On or before 25^th^ November 2014 | | CFR After 25th November 2014 | |
| --- | --- | --- | --- | --- |
|  | Fever Median (%) (95% CI) | No Fever Median (%) (95% CI) | Fever Median (%) (95% CI) | No Fever Median (%) (95% CI) |
| confirmed, probable and suspected cases | 70.2 (69.1-71.4) | 75.6 (73.0-78.0) | 76.1 (74.6-77.5) | 95.6 (95.0-96.6) |
| confirmed and probable cases | 66.4 (65.0-67.7) | 66.6 (63.0-70.0) | 62.7 (60.3-64.9) | 84.2 (80.2-87.7) |
| confirmed cases | 63.9 (62.3-65.4) | 63.7 (59.8-67.6) | 58.6 (56.1-61.1) | 78.0 (72.4-82.9) |

##

## 2.3 CFR without imputation for individual predictors

We report the CFR without imputation for predictors in the model (Table S3).

Table S3: CFR estimates without imputation for candidate predictors.

| Predictors | CFR without imputation for individual predictors | | |
| --- | --- | --- | --- |
|  | Confirmed cases Median (95% CI) | Confirmed and probable cases Median (95% CI) | Confirmed, probable and suspected cases Median (95% CI) |
| Gender | | | |
| male | 68.1 (66.7-69.4) | 70.6 (69.4-71.8) | 78.7 (77.9-79.5) |
| female | 64.1 (62.7-65.4) | 67.1 (65.9-68.3) | 76.0 (75.1-76.9) |
| Health Care Worker |  |  |  |
| Health Care Worker Position | | | |
| High contact | 61.7 (56.1-67.2) | 63.0 (57.6-68.1) | 65.7 (60.8-70.4) |
| Mid contact | 60.6 (52.8-68.0) | 61.5 (54.1-68.6) | 62.3 (55.2-68.9) |
| Low contact | 84.8 (68.1-94.9) | 82.1 (66.5-92.5) | 80.4 (66.9-90.2) |
| Other | 60.3 (47.2-72.4) | 64.9 (53.2-75.5) | 71.7 (61.8-80.3) |
| Bleeding Gums | 80.0 (67.7-89.2) | 82.6 (72.9-89.9) | 82.6 (74.7-88.9) |
| Bleeding Nose | 87.8 (73.8-95.9) | 91.2 (80.7-97.1) | 90.1 (81.5-95.6) |
| Bleeding Skin | 100 (59.0-100.0) | 100 (63.1-100.0) | 92.9 (66.1-99.8) |
| Bleeding Stool | 75.0 (65.1-83.3) | 78.6 (69.8-85.8) | 79.4 (71.4-86.0) |
| Bleeding Urine | 75.0 (47.6-92.7) | 70.6 (44.0-89.7) | 69.6 (47.1-86.8) |
| Haematemesis | 72.7 (59.0-83.9) | 78.6 (67.1-87.5) | 79.8 (70.2-87.4) |
| Vomiting | 62.9 (61.3-64.5) | 66.6 (65.2-68.0) | 70.3 (69.1-71.4) |
| Cough | 66.0 (63.2-68.8) | 68.5 (66.0-70.9) | 72.6 (70.6-74.5) |
| Bleeding Other | 70.8 (58.2-81.4) | 74.4 (63.2-83.6) | 78.4 (69.2-86.0) |
| Bleeding Injection site | 78.9 (62.7-90.4) | 83.7 (70.3-92.7) | 84.2 (72.1-92.5) |
| Bleeding Vagina | 84.0 (63.9-95.5) | 81.8 (64.5-93.0) | 78.8 (65.3-88.9) |
| Unexplained Bleeding | 70.7 (66.5-74.5) | 75.3 (71.8-78.6) | 78.2 (75.3-80.9) |
| Fever | 62.7 (61.4-63.9) | 65.7 (64.6-66.8) | 72.5 (71.7-73.4) |
| Jaundice | 67.5 (62.4-72.2) | 69.6 (65.3-73.7) | 73.7 (70.2-76.9) |
| Painful Eyes | 65.8 (58.9-72.4) | 68.3 (62.4-73.8) | 70.8 (65.7-75.5) |
| Rash | 73.2 (66.7-79.1) | 74.5 (69.0-79.5) | 79.3 (75.3-82.9) |
| Unconsciousness | 80.6 (72.6-87.2) | 83.8 (77.6-88.9) | 87.4 (83.0-91.0) |
| Fatigue | 62.3 (60.9-63.6) | 65.5 (64.3-66.7) | 72.4 (71.5-73.3) |
| Anorexia | 62.3 (60.9-63.7) | 65.4 (64.1-66.7) | 72.2 (71.2-73.2) |
| Vomiting | 62.9 (61.3-64.5) | 66.6 (65.2-68.0) | 70.3 (69.1-71.4) |
| Diarrhoea | 64.3 (62.7-65.9) | 67.8 (66.4-69.2) | 71.1 (69.8-72.3) |
| Headache | 61.6 (60.0-63.2) | 64.7 (63.2-66.1) | 71.2 (70.1-72.3) |
| Abdominal Pain | 63.9 (62.1-65.6) | 66.7 (65.2-68.3) | 73.6 (72.4-74.7) |
| Muscle Pain | 63.9 (62.1-65.6) | 66.8 (65.2-68.4) | 72.9 (71.7-74.1) |
| Joint Pain | 63.5 (61.7-65.3) | 66.5 (65.0-68.1) | 73.2 (71.9-74.3) |
| Chest Pain | 64.9 (62.5-67.3) | 67.6 (65.5-69.6) | 72.2 (70.5-73.8) |
| Coughing Blood | 66.0 (63.2-68.8) | 68.5 (66.0-70.9) | 72.6 (70.6-74.5) |
| Difficult Breathing | 74.8 (72.2-77.2) | 77.9 (75.7-79.9) | 84.1 (82.8-85.4) |
| Conjunctivitis | 67.2 (64.5-69.9) | 68.0 (65.4-70.4) | 71.3 (69.2-73.4) |
| Sore Throat | 70.9 (67.2-74.4) | 72.6 (69.4-75.6) | 75.2 (72.6-77.7) |
| Confused | 73.8 (68.8-78.4) | 75.8 (71.5-79.8) | 80.6 (77.3-83.5) |
| Hiccup | 72.4 (68.8-75.9) | 75.3 (72.1-78.3) | 78.9 (76.4-81.2) |
| Difficulty Swallowing | 72.0 (69.2-74.6) | 74.2 (71.8-76.4) | 78.8 (77.0-80.5) |
|  |  |  |  |

## 2.4 Proportion of predictors with known and unknown survival outcomes

We report the missingness in the predictors used this analysis for confirmed, probable and suspected cases, confirmed and probable and confirmed cases. The BRT model, unlike many other algorithms (e.g. random forest), inherently handles this missingness in predictors when creating the trees and hence imputation of the predictors was not required. Briefly, missingness in each predictor is treated as a dummy predictor level and each non-terminal node is assigned three child nodes, one for the missing values and the other two defined by the threshold used to split on the predictor values with complete (non-missing) data.

**Confirmed, probable and suspected cases**

Table S4: Proportion of missingness in candidate predictors for confirmed, probable and suspected cases.

| Predictors | Known Survival Outcomes | | | Unknown Survival Outcomes | | |
| --- | --- | --- | --- | --- | --- | --- |
|  | Non-missing Cases | Missing Cases | % Missingness | Non-missing Cases | Missing Cases | % Missingness |
| Bleeding Gums | 5326 | 13318 | 71.4 | 3296 | 11398 | 77.6 |
| Bleeding Nose | 5310 | 13334 | 71.5 | 3280 | 11414 | 77.7 |
| Bleeding Skin | 5274 | 13370 | 71.7 | 3254 | 11440 | 77.9 |
| Bleeding Stool | 5320 | 13324 | 71.5 | 3279 | 11415 | 77.7 |
| Bleeding Urine | 5271 | 13373 | 71.7 | 3250 | 11444 | 77.9 |
| Hematemesis | 5123 | 13521 | 72.5 | 3207 | 11487 | 78.2 |
| Bloody Vomit | 5091 | 13553 | 72.7 | 3185 | 11509 | 78.3 |
| Bloody Cough | 5280 | 13364 | 71.7 | 3256 | 11438 | 77.8 |
| Bleeding Other | 5168 | 13476 | 72.3 | 3215 | 11479 | 78.1 |
| Bleeding Injection site | 5297 | 13347 | 71.6 | 3257 | 11437 | 77.8 |
| Bleeding Vagina | 5190 | 13454 | 72.2 | 3212 | 11482 | 78.1 |
| Unexplained bleeding | 11580 | 7064 | 37.9 | 7976 | 6718 | 45.7 |
| Fever | 14010 | 4634 | 24.9 | 10238 | 4456 | 30.3 |
| Fatigue | 13736 | 4908 | 26.3 | 10037 | 4657 | 31.7 |
| Anorexia | 13255 | 5389 | 28.9 | 9937 | 4757 | 32.4 |
| Vomiting | 13126 | 5518 | 29.6 | 9508 | 5186 | 35.3 |
| Diarrhoea | 12948 | 5696 | 30.6 | 9370 | 5324 | 36.2 |
| Headache | 12675 | 5969 | 32.0 | 9455 | 5239 | 35.7 |
| Abdominal Pain | 12482 | 6162 | 33.1 | 9295 | 5399 | 36.7 |
| Muscle Pain | 12477 | 6167 | 33.1 | 9368 | 5326 | 36.2 |
| Joint Pain | 12309 | 6335 | 34.0 | 9404 | 5290 | 36.0 |
| Chest Pain | 9289 | 9355 | 50.2 | 6502 | 8192 | 55.8 |
| Cough | 9047 | 9597 | 51.5 | 6262 | 8432 | 57.4 |
| Difficult Breathing | 11497 | 7147 | 38.3 | 8757 | 5937 | 40.4 |
| Difficult Swallowing | 11125 | 7519 | 40.3 | 8573 | 6121 | 41.7 |
| Conjunctivitis | 11134 | 7510 | 40.3 | 8669 | 6025 | 41.0 |
| Sore Throat | 8822 | 9822 | 52.7 | 6350 | 8344 | 56.8 |
| Confused | 4975 | 13669 | 73.3 | 3202 | 11492 | 78.2 |
| Hiccups | 11558 | 7086 | 38.0 | 8368 | 6326 | 43.1 |
| Jaundice | 5056 | 13588 | 72.9 | 3288 | 11406 | 77.6 |
| Painful Eyes | 4878 | 13766 | 73.8 | 3154 | 11540 | 78.5 |
| Rash | 9747 | 8897 | 47.7 | 7788 | 6906 | 47.0 |
| Unconsciousness | 4921 | 13723 | 73.6 | 3151 | 11543 | 78.6 |
| Districts in Sierra Leone | 18358 | 286 | 1.5 | 13969 | 725 | 4.9 |
| District in Liberia | 18358 | 286 | 1.5 | 13969 | 725 | 4.9 |
| Districts in Guinea | 18358 | 286 | 1.5 | 13969 | 725 | 4.9 |
| Gender | 18096 | 548 | 2.9 | 14372 | 322 | 2.2 |
| Age | 18171 | 473 | 2.5 | 13629 | 1065 | 7.2 |
| Quarter | 16610 | 2034 | 10.9 | 10730 | 3964 | 27.0 |
| Health care workers | 18644 | - | - | 14694 | - | - |
| Hospitalisation Status | 14811 | 3833 | 20.6 | 9457 | 5237 | 35.6 |
| Case Definition | 18644 | - | - | 14694 | - | - |
| Health care worker position | 748 | 17896 | 96.0 | 284 | 14410 | 98.1 |
| Delay | 14918 | 3726 | 20.0 | 9699 | 4995 | 34.0 |

**Confirmed and probable cases**

Table S5: Proportion of missingness in candidate predictors for confirmed and probable cases.

| Predictors | Known Survival Outcomes | | | Unknown Survival Outcomes | | |
| --- | --- | --- | --- | --- | --- | --- |
|  | Non-missing Cases | Missing Cases | % Missingness | Non-missing Cases | Missing Cases | % Missingness |
| Bleeding Gums | 3932 | 7360 | 65.2 | 2260 | 6802 | 75.1 |
| Bleeding Nose | 3923 | 7369 | 65.3 | 2252 | 6810 | 75.1 |
| Bleeding Skin | 3895 | 7397 | 65.5 | 2237 | 6825 | 75.3 |
| Bleeding Stool | 3936 | 7356 | 65.1 | 2250 | 6812 | 75.2 |
| Bleeding Urine | 3897 | 7395 | 65.5 | 2239 | 6823 | 75.3 |
| Hematemesis | 3744 | 7548 | 66.8 | 2186 | 6876 | 75.9 |
| Blood Vomiting | 3717 | 7575 | 67.1 | 2178 | 6884 | 76.0 |
| Bloody Cough | 3901 | 7391 | 65.5 | 2238 | 6824 | 75.3 |
| Bleeding Other | 3778 | 7514 | 66.5 | 2193 | 6869 | 75.8 |
| Bleeding Injection site | 3915 | 7377 | 65.3 | 2242 | 6820 | 75.3 |
| Bleeding Vagina | 3838 | 7454 | 66.0 | 2209 | 6853 | 75.6 |
| Unexplained bleeding | 6054 | 5238 | 46.4 | 4291 | 4771 | 52.6 |
| Fever | 8021 | 3271 | 29.0 | 5619 | 3443 | 38.0 |
| Fatigue | 7808 | 3484 | 30.9 | 5496 | 3566 | 39.4 |
| Anorexia | 7342 | 3950 | 35.0 | 5443 | 3619 | 39.9 |
| Vomiting | 7286 | 4006 | 35.5 | 5226 | 3836 | 42.3 |
| Diarrhoea | 7169 | 4123 | 36.5 | 5161 | 3901 | 43.0 |
| Headache | 6987 | 4305 | 38.1 | 5198 | 3864 | 42.6 |
| Abdominal Pain | 6749 | 4543 | 40.2 | 5067 | 3995 | 44.1 |
| Muscle Pain | 6795 | 4497 | 39.8 | 5101 | 3961 | 43.7 |
| Joint Pain | 6640 | 4652 | 41.2 | 5115 | 3947 | 43.6 |
| Chest Pain | 5011 | 6281 | 55.6 | 3588 | 5474 | 60.4 |
| Cough | 4840 | 6452 | 57.1 | 3444 | 5618 | 62.0 |
| Difficult Breathing | 5740 | 5552 | 49.2 | 4688 | 4374 | 48.3 |
| Difficult Swallowing | 5486 | 5806 | 51.4 | 4593 | 4469 | 49.3 |
| Conjunctivitis | 5611 | 5681 | 50.3 | 4717 | 4345 | 47.9 |
| Sore Throat | 4555 | 6737 | 59.7 | 3455 | 5607 | 61.9 |
| Confused | 3571 | 7721 | 68.4 | 2225 | 6837 | 75.4 |
| Hiccups | 5988 | 5304 | 47.0 | 4508 | 4554 | 50.3 |
| Jaundice | 3634 | 7658 | 67.8 | 2264 | 6798 | 75.0 |
| Painful Eyes | 3518 | 7774 | 68.8 | 2206 | 6856 | 75.7 |
| Rash | 5032 | 6260 | 55.4 | 4204 | 4858 | 53.6 |
| Unconscious | 3545 | 7747 | 68.6 | 2197 | 6865 | 75.8 |
| Districts in Sierra Leone | 11065 | 227 | 2.0 | 8519 | 543 | 6.0 |
| Districts in Liberia | 11065 | 227 | 2.0 | 8519 | 543 | 6.0 |
| Districts in Guinea | 11065 | 227 | 2.0 | 8519 | 543 | 6.0 |
| Gender | 11237 | 55 | 0.5 | 8882 | 180 | 2.0 |
| Age | 11023 | 269 | 2.4 | 8353 | 709 | 7.8 |
| Quarter | 10090 | 1202 | 10.6 | 5874 | 3188 | 35.2 |
| Health care workers | 11292 | - | - | 9062 | - | - |
| Hospitalisation status | 9222 | 2070 | 18.3 | 5440 | 3622 | 40.0 |
| Case definition | 11292 | - | - | 9062 | - | - |
| Health care worker position | 641 | 10651 | 94.3 | 177 | 8885 | 98.0 |
| Delay | 9617 | 1675 | 14.8 | 5356 | 3706 | 40.9 |

**Confirmed cases**

Table S6: Proportion of missingness in candidate predictors for confirmed cases.

| Predictors | Known Survival Outcomes | | | Unknown Survival Outcomes | | |
| --- | --- | --- | --- | --- | --- | --- |
|  | Non-missing Cases | Missing Cases | % Missingness | Non-missing Cases | Missing Cases | % Missingness |
| Bleeding Gums | 3063 | 6408 | 67.7 | 1333 | 5640 | 80.9 |
| Bleeding Nose | 3057 | 6414 | 67.7 | 1328 | 5645 | 81.0 |
| Bleeding Skin | 3038 | 6433 | 67.9 | 1314 | 5659 | 81.2 |
| Bleeding Stool | 3073 | 6398 | 67.6 | 1322 | 5651 | 81.0 |
| Bleeding Urine | 3039 | 6432 | 67.9 | 1319 | 5654 | 81.1 |
| Hematemesis | 2876 | 6595 | 69.6 | 1264 | 5709 | 81.9 |
| Blood Vomiting | 2857 | 6614 | 69.8 | 1256 | 5717 | 82.0 |
| Blood Cough | 3041 | 6430 | 67.9 | 1317 | 5656 | 81.1 |
| Bleeding Other | 2906 | 6565 | 69.3 | 1269 | 5704 | 81.8 |
| Bleeding Inject site | 3052 | 6419 | 67.8 | 1320 | 5653 | 81.1 |
| Bleeding Vagina | 2995 | 6476 | 68.4 | 1295 | 5678 | 81.4 |
| Unexplained bleeding | 4766 | 4705 | 49.7 | 2818 | 4155 | 59.6 |
| Fever | 6523 | 2948 | 31.1 | 3862 | 3111 | 44.6 |
| Fatigue | 6336 | 3135 | 33.1 | 3770 | 3203 | 45.9 |
| Anorexia | 5903 | 3568 | 37.7 | 3730 | 3243 | 46.5 |
| Vomiting | 5843 | 3628 | 38.3 | 3533 | 3440 | 49.3 |
| Diarrhoea | 5734 | 3737 | 39.5 | 3490 | 3483 | 49.9 |
| Headache | 5591 | 3880 | 41.0 | 3532 | 3441 | 49.3 |
| Abdominal Pain | 5401 | 4070 | 43.0 | 3431 | 3542 | 50.8 |
| Muscle Pain | 5440 | 4031 | 42.6 | 3465 | 3508 | 50.3 |
| Joint Pain | 5292 | 4179 | 44.1 | 3477 | 3496 | 50.1 |
| Chest Pain | 3942 | 5529 | 58.4 | 2247 | 4726 | 67.8 |
| Cough | 3784 | 5687 | 60.0 | 2134 | 4839 | 69.4 |
| Difficult Breathing | 4524 | 4947 | 52.2 | 3100 | 3873 | 55.5 |
| Difficult Swallowing | 4299 | 5172 | 54.6 | 3005 | 3968 | 56.9 |
| Conjunctivitis | 4444 | 5027 | 53.1 | 3130 | 3843 | 55.1 |
| Sore Throat | 3528 | 5943 | 62.7 | 2135 | 4838 | 69.4 |
| Confused | 2761 | 6710 | 70.8 | 1278 | 5695 | 81.7 |
| Hiccups | 4693 | 4778 | 50.4 | 2952 | 4021 | 57.7 |
| Jaundice | 2820 | 6651 | 70.2 | 1308 | 5665 | 81.2 |
| Painful Eyes | 2713 | 6758 | 71.4 | 1261 | 5712 | 81.9 |
| Rash | 3933 | 5538 | 58.5 | 2770 | 4203 | 60.3 |
| Unconsciousness | 2731 | 6740 | 71.2 | 1251 | 5722 | 82.1 |
| Districts in Sierra Leone | 9260 | 211 | 2.2 | 6478 | 495 | 7.1 |
| Districts in Liberia | 9260 | 211 | 2.2 | 6478 | 495 | 7.1 |
| Districts in Guinea | 9260 | 211 | 2.2 | 6478 | 495 | 7.1 |
| Gender | 9428 | 43 | 0.5 | 6827 | 146 | 2.1 |
| Age | 9246 | 225 | 2.4 | 6356 | 617 | 8.8 |
| Quarter | 8397 | 1074 | 11.3 | 4212 | 2761 | 39.6 |
| Health care workers | 9471 | - | - | 6973 | - | - |
| Hospitalisation status | 7821 | 1650 | 17.4 | 3864 | 3109 | 44.6 |
| Case definition | 9471 | - | - | 6973 | - | - |
| Health care worker position | 577 | 8894 | 93.9 | 128 | 6845 | 98.2 |
| Delay | 8010 | 1461 | 15.4 | 3793 | 3180 | 45.6 |

##

## 2.5 Identification of hyperparameters.

We created 72 hyperparameter combinations from tree complexities varying from 5 to 40 in steps of 5, learning rates of 0.01,0.001 and 0.0005 and bag fractions from 0.5 to 1 in steps of 0.25. Additionally, to optimise the data partitioning for model training and validation, partitioning ratios of 0.5, 0.65 and 0.8.

Figure S1 shows prediction accuracy on the validation data which is a measure of out-of-sample predictive performance and Figure S2 shows the prediction accuracy on the training data which is a measure of the goodness-of-fit.

We found similar predictive performance measures (sensitivity, specificity, PCC and AUC) for p=0.5 and p=0.8, with slight increase for p=0.65 and that the predictive performance (i) increased with increasing values of tree complexity but tended to level-off beyond tree complexity=27, (ii) was highest for learning rates of 0.001 and lowest for learning rate of 0.0005, (iii) was consistently higher for bag fraction 0.5 and 0.75 regardless of the partitioning ratio (Figure S1).


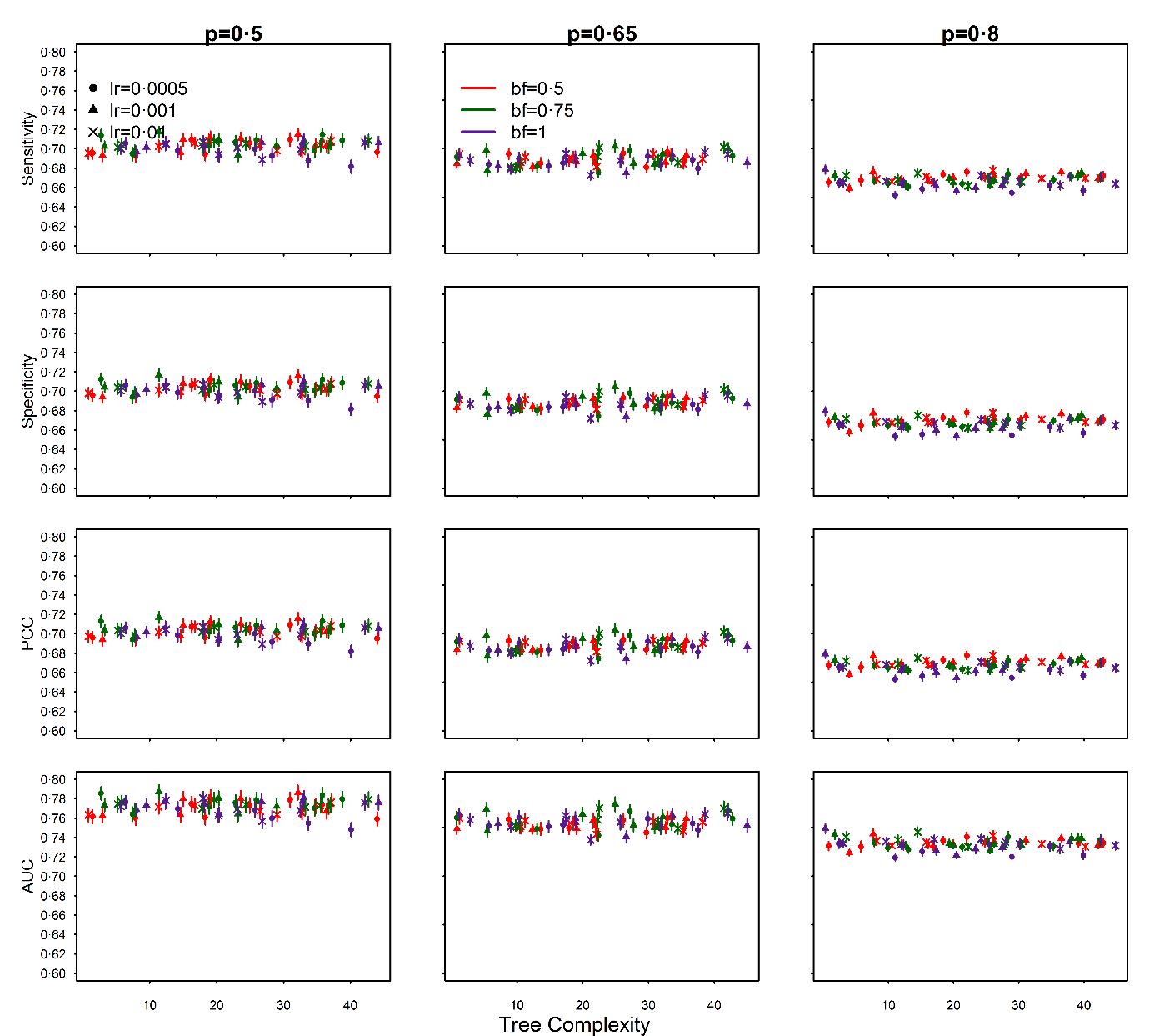


Figure S1: Out-of-sample predictive accuracy measures (sensitivity, specificity, proportion of predictions correctly classified (PCC) and area under the receiver operating curve (AUC)) on the validation data obtained using 72 different hyperparameterisations of the BRT model. p represents the proportion of data to train the model with p=0.5, p=0.65 and p=0.8 explored.


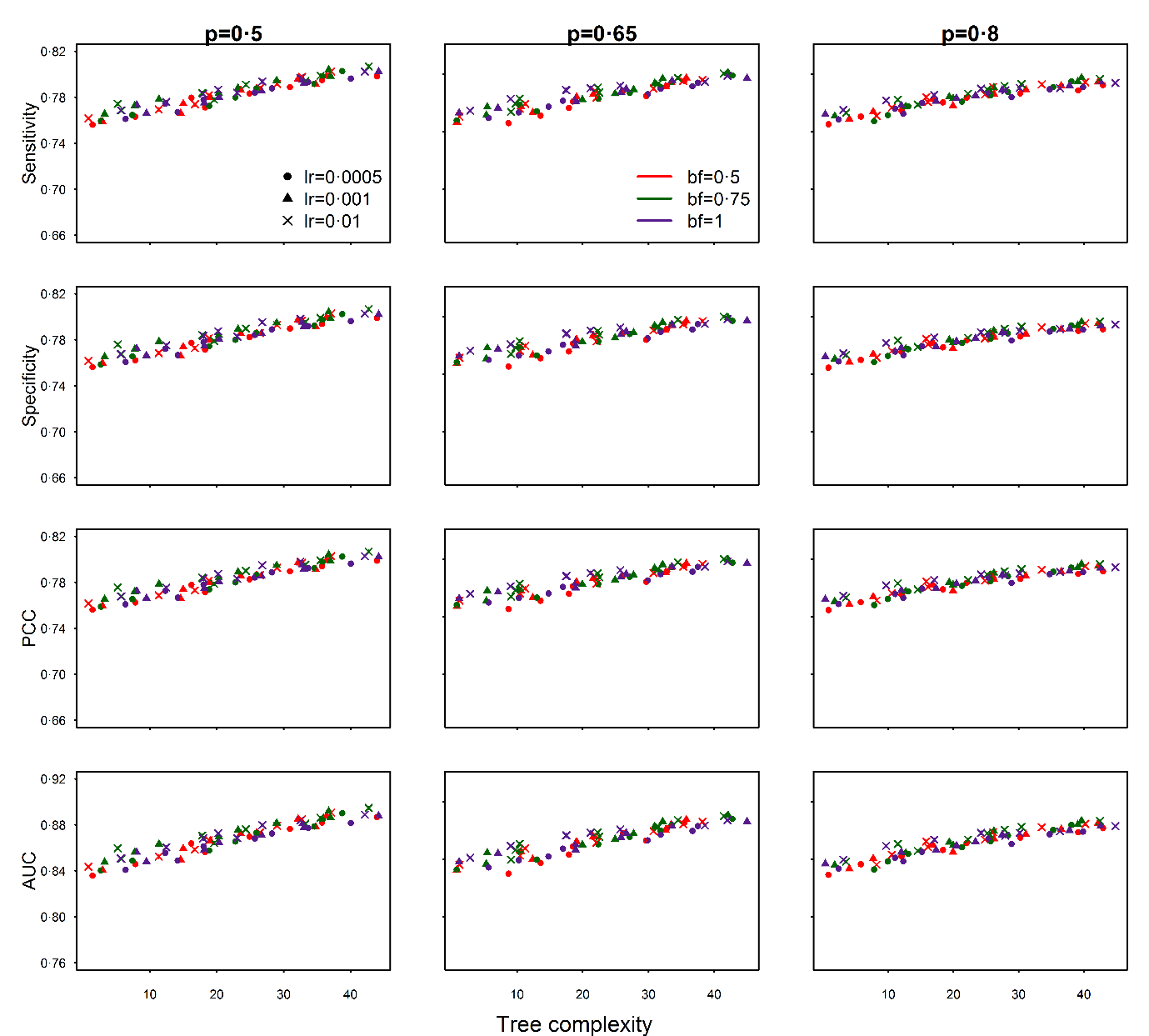


Figure S2: Goodness-of-fit accuracy measures (sensitivity, specificity, proportion of predictions correctly classified (PCC) and area under the receiver operating curve (AUC)) on the training data obtained using 72 different hyperparameterisations of the BRT model. p represents the proportion of data to train the model with p=0.5, p=0.65 and p=0.8 explored.

## 2.6 Robustness of BRT imputation to data down sampling

In our main analysis, we used the complete line list data to impute unknown survival outcomes. By the end of the West African Ebola epidemic, the data to which we fitted the BRT model was large relative to typical epidemiological datasets in outbreak settings. Subsequent outbreaks will provide smaller datasets especially at the initial stages. To investigate the robustness of BRT modelling to different outbreak sample sizes, we down sampled our dataset from 100% of cases (as presented in the main analyses) to 75%, 50%, 25% and 10% of cases and estimated the imputation performance in each scenario (Figure S3). It is reassuring that BRT mean performance varied only slightly with decreased sample size. However, as expected the uncertainty surrounding measures of performance increased as sample size decreased. It is important to be able to communicate these types of analyses to policy makers and public health responders in the frontlines of outbreaks as our study show which predictors would be important to obtain reliable estimates in the future.


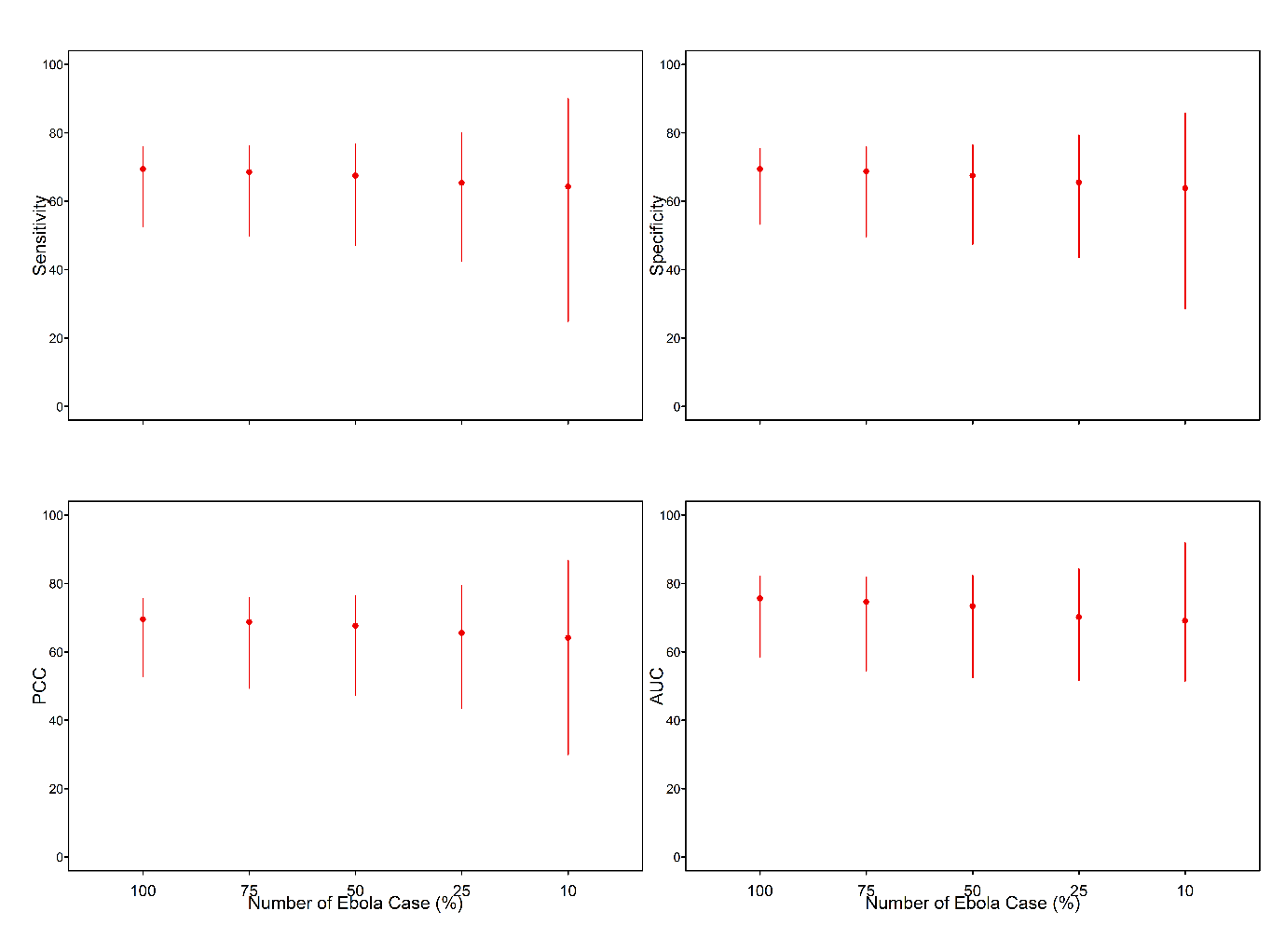


Figure S3: Performance (i.e. sensitivity, specificity, PCC, and AUC) of BRT model after down-sampling of data for all Ebola cases (i.e. confirmed, probable and suspected).

## 2.7 Comparison of CFR estimates obtained on the training set, without and with imputation

The goodness-of-fit performance (i.e. making prediction using the data on which the model was built) on the training data was very good for all predictors and for all case definition combinations as confidence intervals neatly overlap (Figures S4—S9).

### 2.7.1 Confirmed, probable, and suspected cases

#### CFR for age, delay, country, and fever


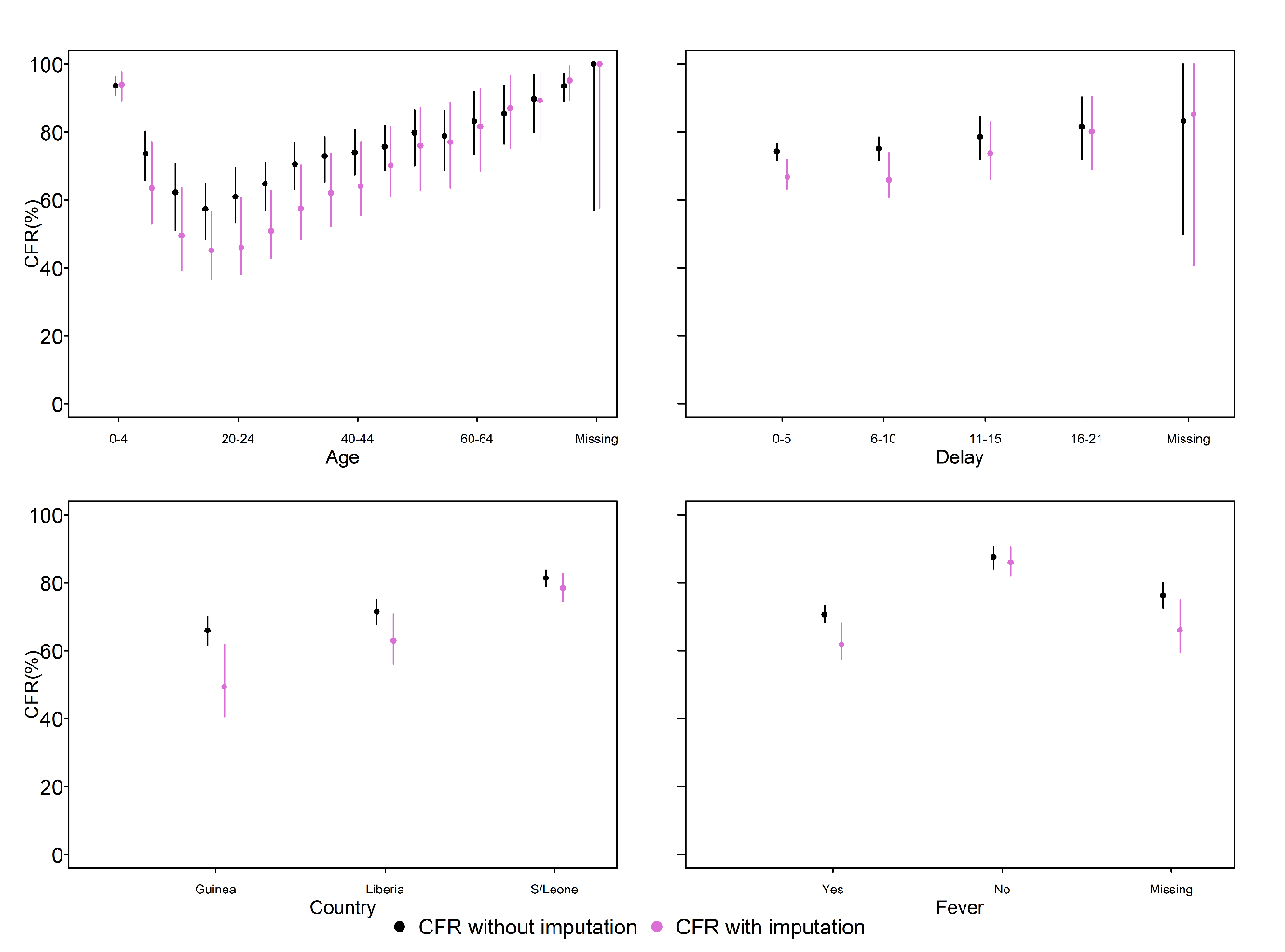


Figure S4: CFR estimates without (black) and with (orchid) imputation for age, delay, country and fever , for confirmed, probable and suspected cases obtained for the training data using the (simplified) BRT model with tree complexity =27, learning rate=0.001, bag fraction=0.75, data partitioning ratio=0.65. Median and 95% confidence intervals (CI) plotted (based on 1,000 bootstrap realisations).

#### CFR for other Predictors


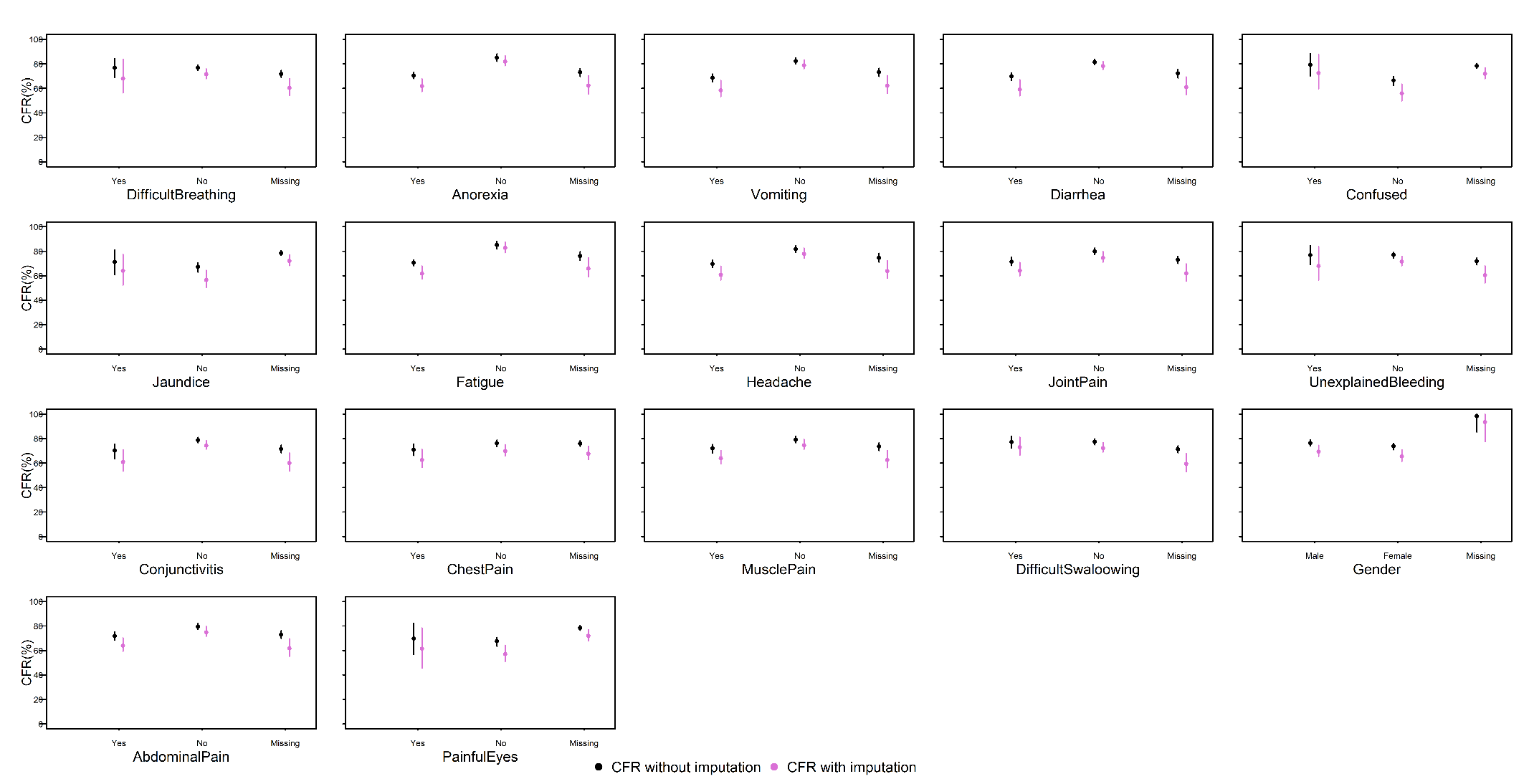


Figure S5: CFR estimates without (black) and without (orchid) imputation for other predictors , for confirmed, probable and suspected cases obtained for the training data using the (simplified) BRT model with tree complexity =27, learning rate=0.001, bag fraction=0.75, data partitioning ratio=0.65. Median and 95% confidence intervals (CI) plotted (based on 1,000 bootstrap realisations).

### 2.7.2 Confirmed and probable cases.

#### CFR for age, delay, country, and fever


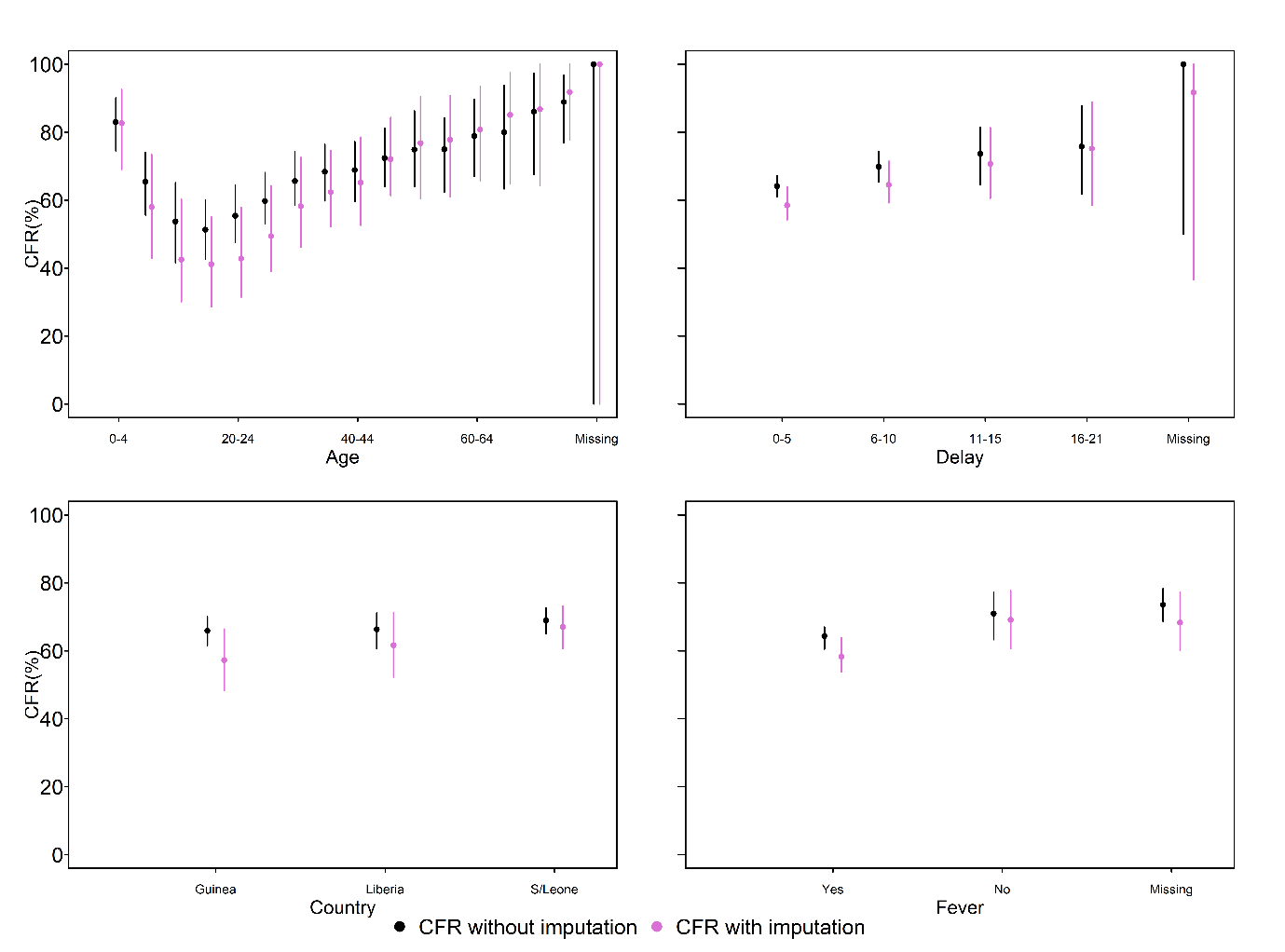


Figure S6: CFR estimates without (black) and with (orchid) imputation for age, delay, country, and fever , for confirmed and probable cases obtained for the training data using the (simplified) BRT model with tree complexity =27, learning rate=0.001, bag fraction=0.75, data partitioning ratio=0.65. Median and 95% confidence intervals (CI) plotted (based on 1,000 bootstrap realisations).

#### CFR for other Predictors


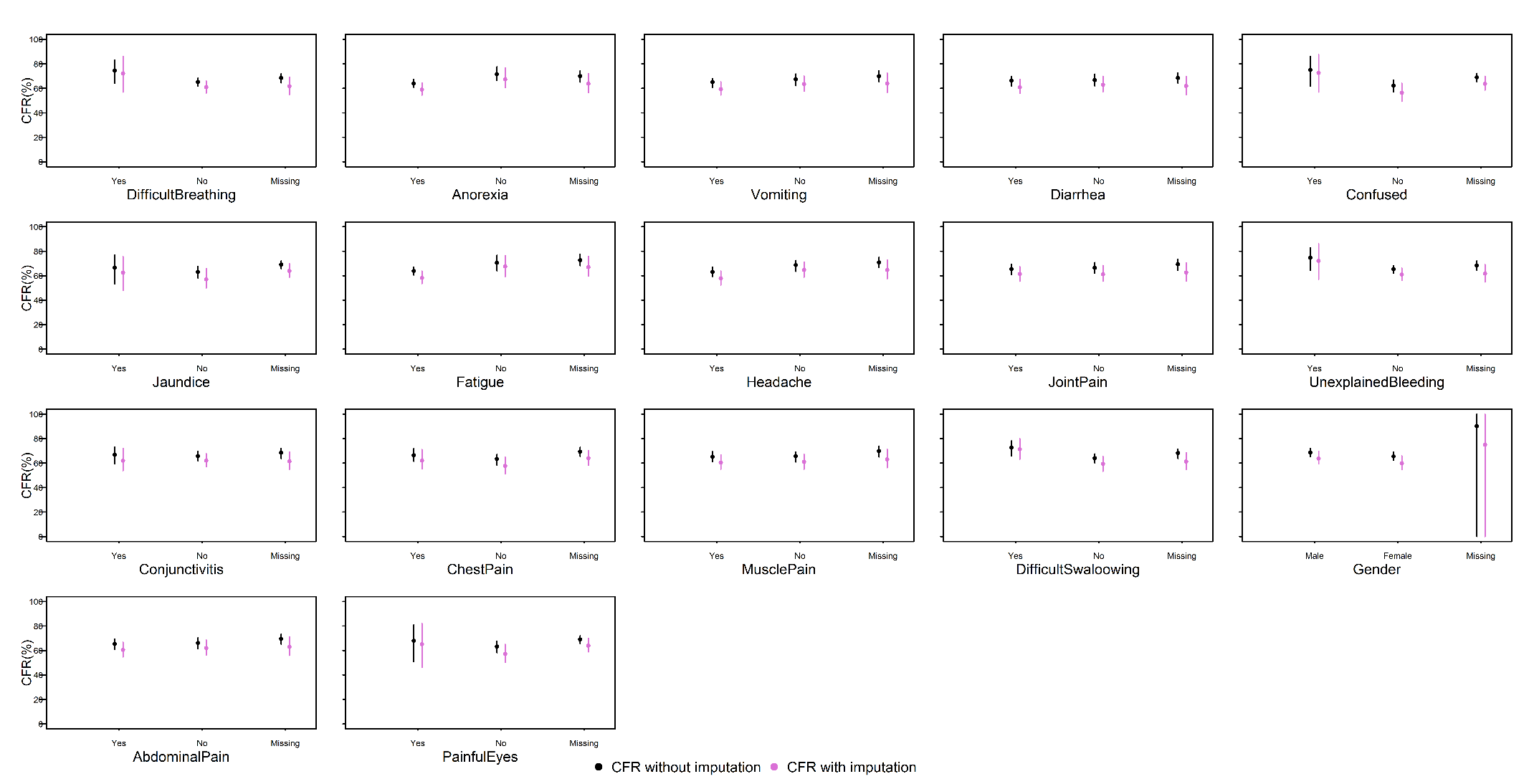


Figure S7: CFR estimates without (black) and with (orchid) imputation for other predictors , for confirmed and probable cases obtained for the training data using the (simplified) BRT model with tree complexity =27, learning rate=0.001, bag fraction=0.75, data partitioning ratio=0.65. Median and 95% confidence intervals (CI) plotted (based on 1,000 bootstrap realisations).

### 2.7.3 Confirmed cases.

#### CFR for age, delay, country, and fever


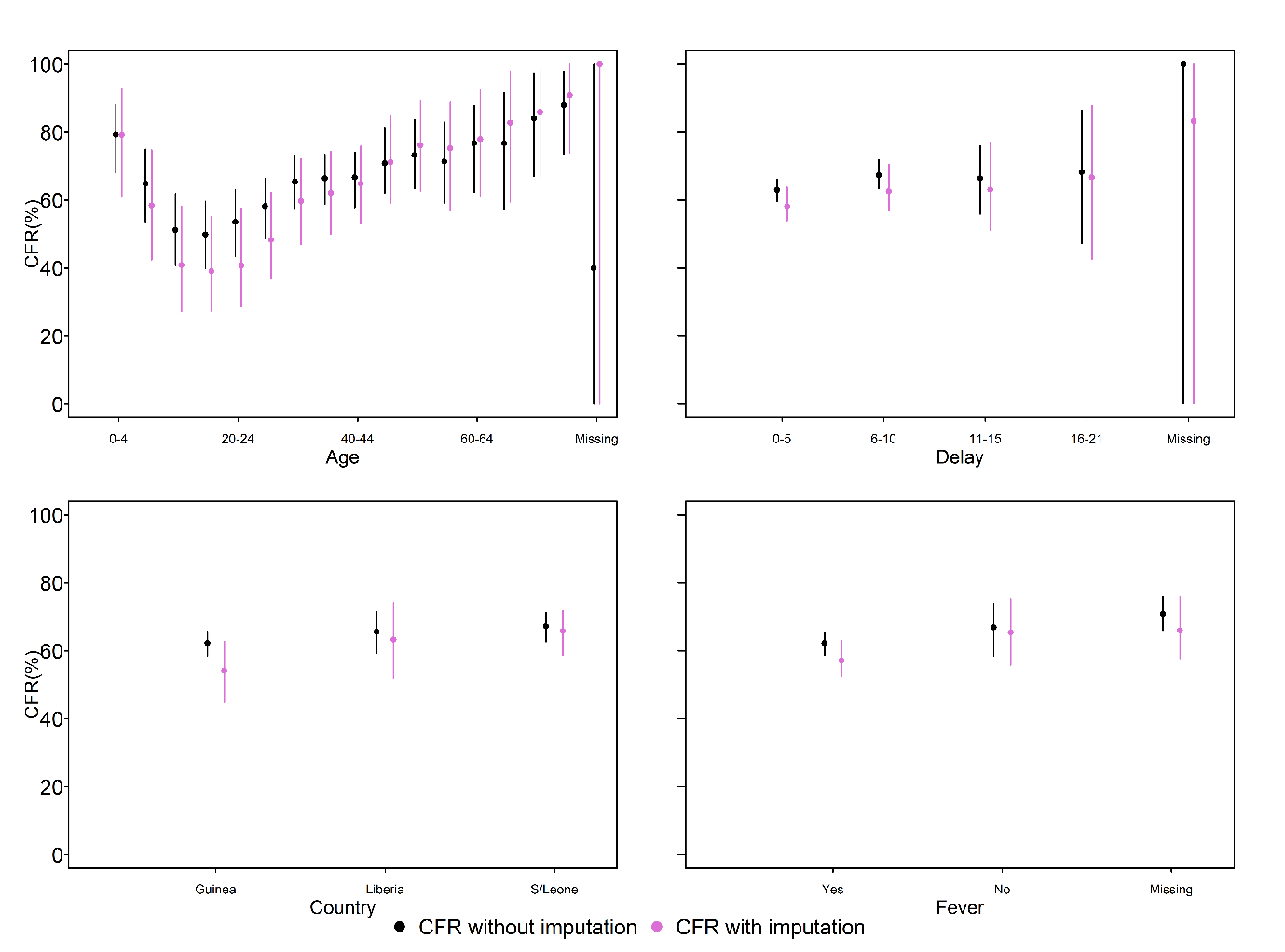


Figure S8: CFR estimates without (black) and with (orchid) imputation for age, delay, country, and fever , for confirmed cases obtained for the training data using the (simplified) BRT model with tree complexity =27, learning rate=0.001, bag fraction=0.75, data partitioning ratio=0.65. Median and 95% confidence intervals (CI) plotted (based on 1,000 bootstrap realisations).

#### CFR for other Predictors


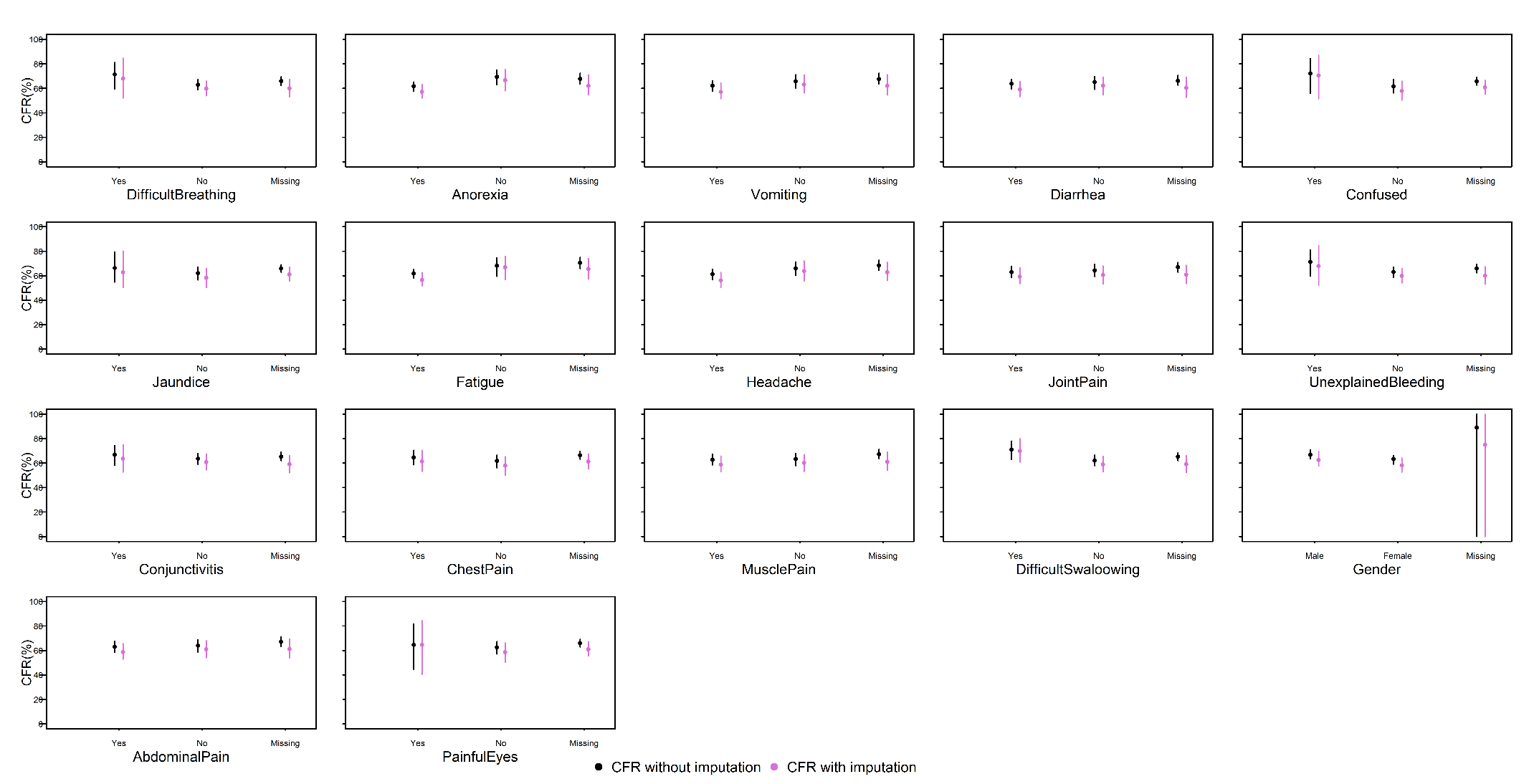


Figure S9: CFR estimates without (black) and with (orchid) imputation for other predictors , for confirmed cases obtained for the training data using the (simplified) BRT model with tree complexity =27, learning rate=0.001, bag fraction=0.75, data partitioning ratio=0.65. Median and 95% confidence intervals (CI) plotted (based on 1,000 bootstrap realisations).

## 2.8 Comparison of CFR estimates obtained on the validation data, with and without imputation

### 2.8.1 Confirmed, probable, and suspected cases

This is the performance on the validation data (without combining the observed and imputed survival outcomes). We applied the model on the out-of-sample data to evaluate performance. The validation performance was appreciable for all predictors and for all case definition combinations as confidence intervals with and without imputation show some overlap (Figures S10— S15).

#### CFR for age, delay, country, and fever


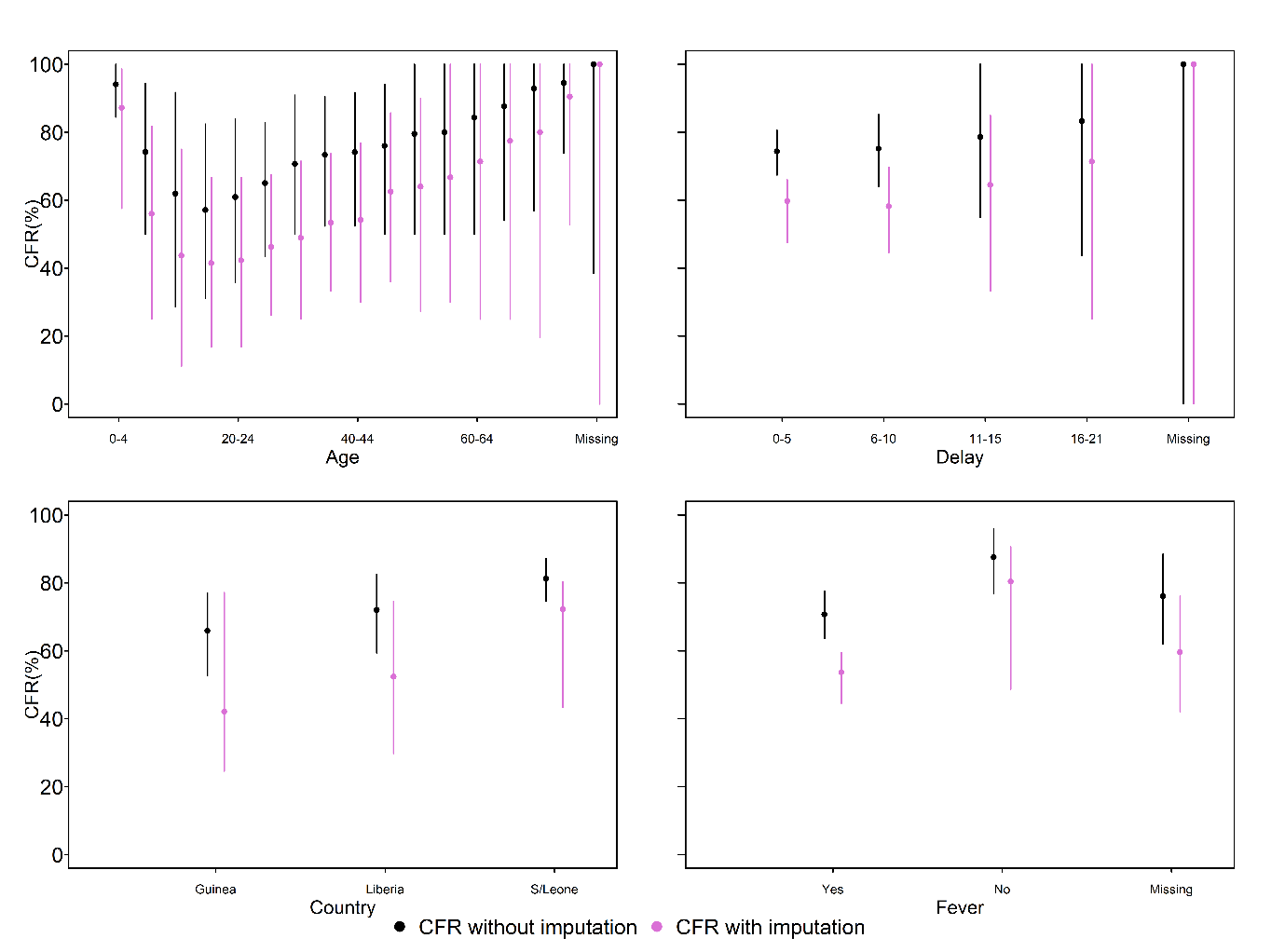


Figure S10: CFR estimates without (black) and with (orchid) imputation for age, delay, country, and fever, for confirmed, probable and suspected cases for the validation data obtained using the (simplified) BRT model with tree complexity =27, learning rate=0.001, bag fraction=0.75, data partitioning ratio=0.65. Median and 95% confidence intervals (CI) plotted (based on 1,000 bootstrap realisations).

#### CFR for other Predictors


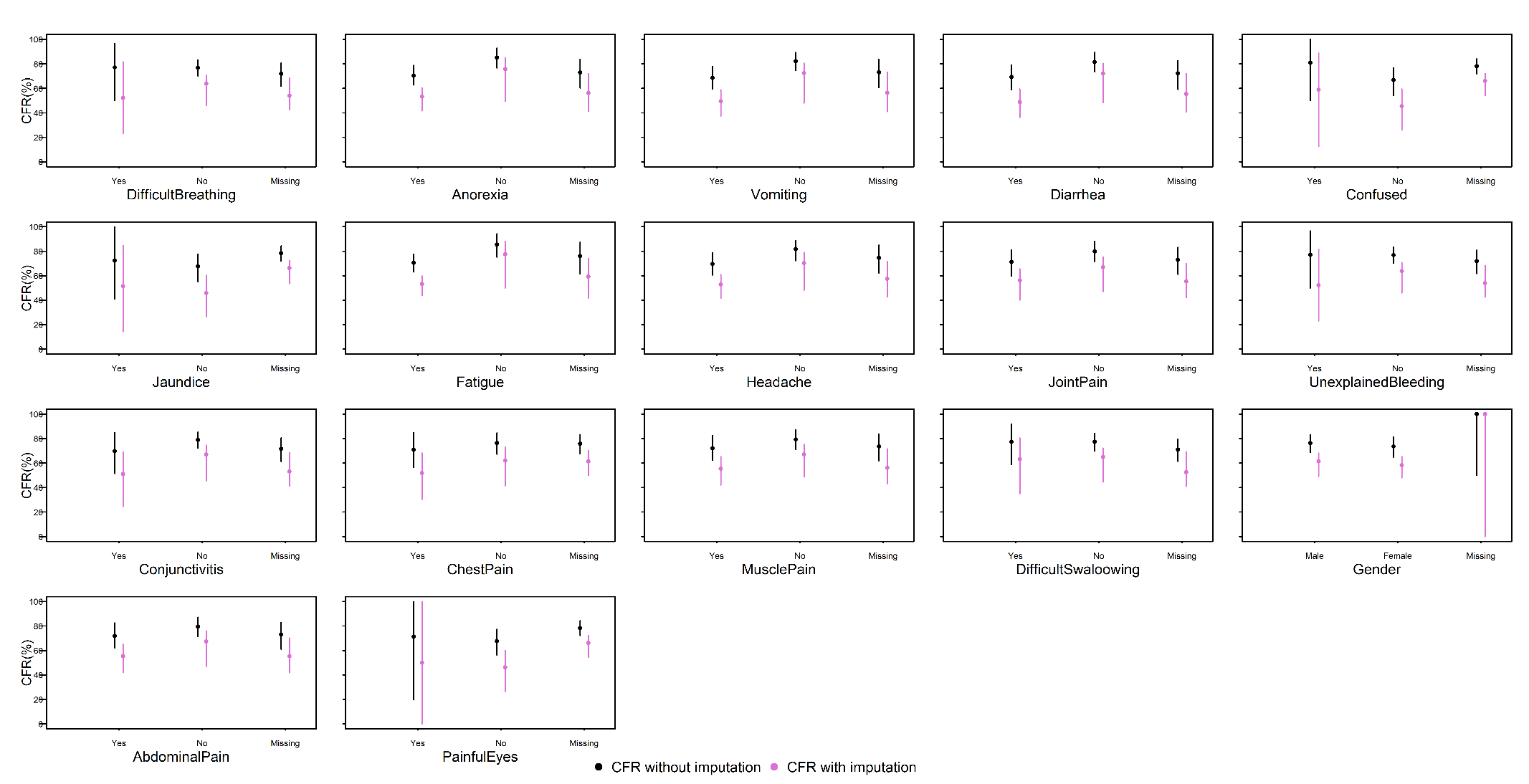


Figure S11: CFR estimates without (black) and with (orchid) imputation for other predictors, for confirmed, probable and suspected cases obtained for the validation data using the (simplified) BRT model with tree complexity =10, learning rate=0.01, bag fraction=0.5, data partitioning ratio=0.8. Median and 95% confidence intervals (CI) plotted (based on 1,000 bootstrap realisations).

### 2.8.2 Confirmed and probable cases

#### CFR for age, delay, country, and fever


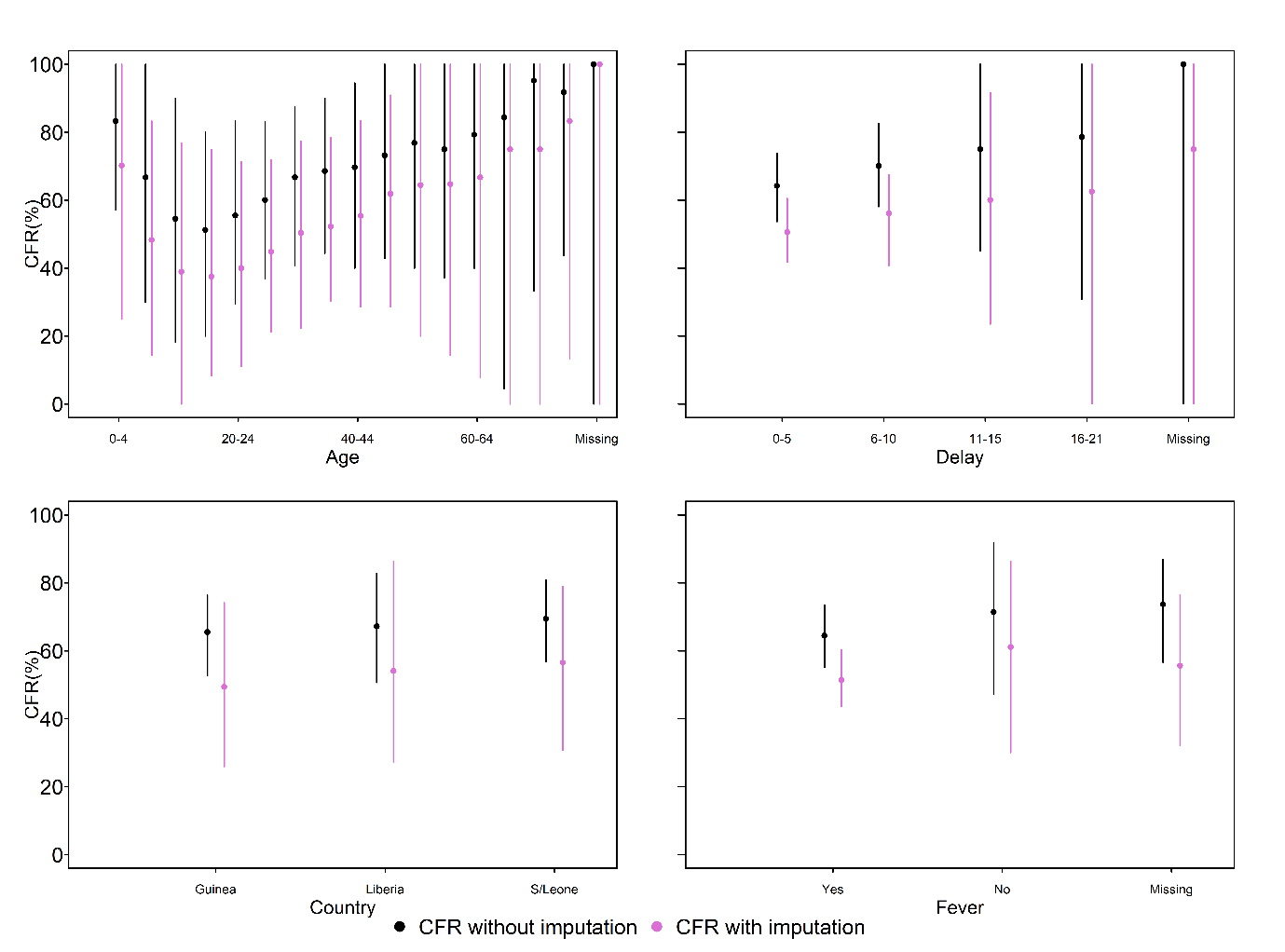


Figure S12: CFR estimates without (black) and with (orchid) imputation for age, delay, country, and fever, for confirmed and probable cases for the validation data obtained using the (simplified) BRT model with tree complexity =27, learning rate=0.001, bag fraction=0.75, data partitioning ratio=0.65. Median and 95% confidence intervals (CI) plotted (based on 1,000 bootstrap realisations).

#### CFR for other Predictors


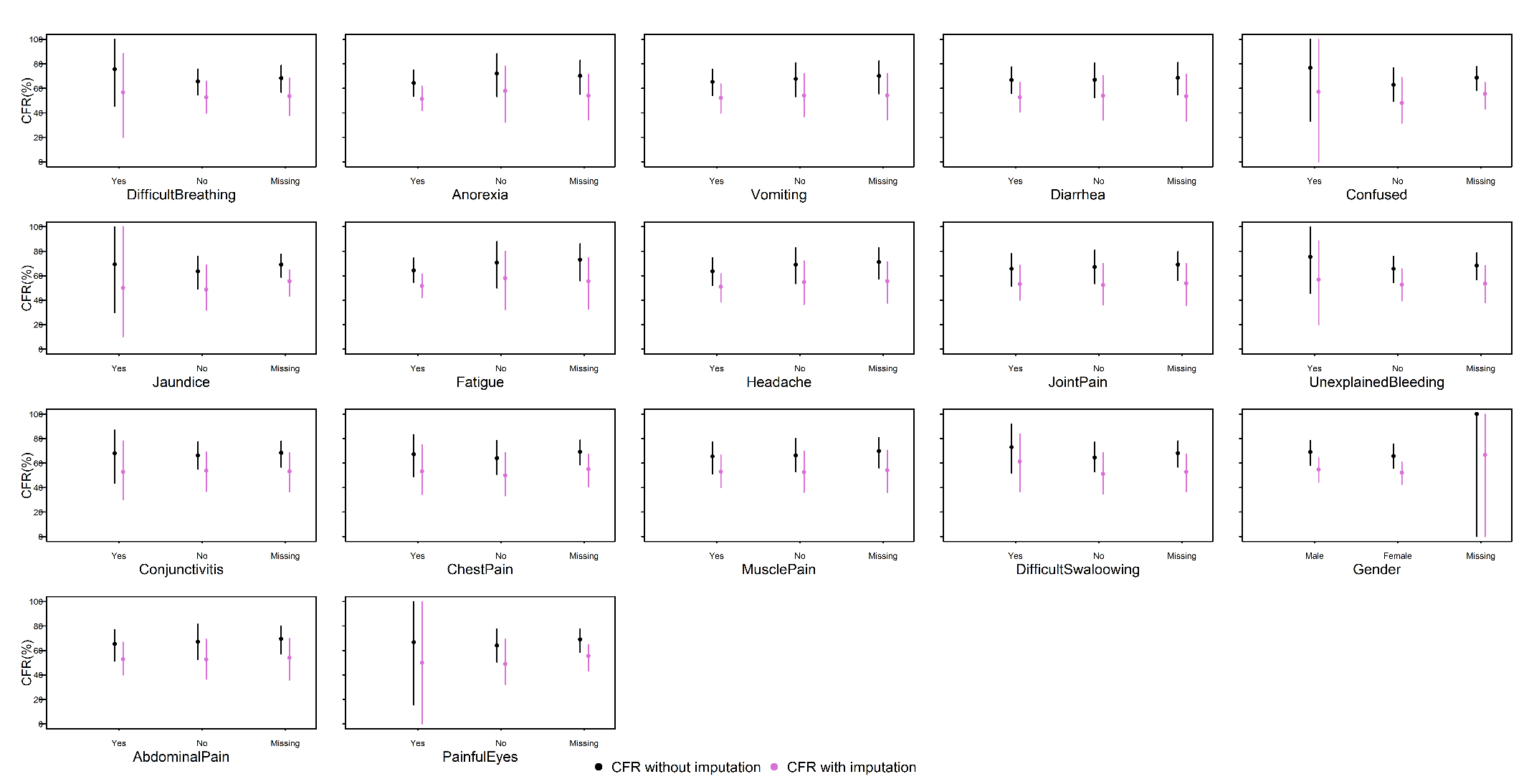


Figure S13: CFR estimates without (black) and with (orchid) imputation for other predictors, for confirmed and probable cases obtained for the validation data using the (simplified) BRT model with tree complexity =10, learning rate=0.01, bag fraction=0.5, data partitioning ratio=0.8. Median and 95% confidence intervals (CI) plotted (based on 1,000 bootstrap realisations).

### 2.8.3 Confirmed cases

#### CFR for age, delay, country, and fever


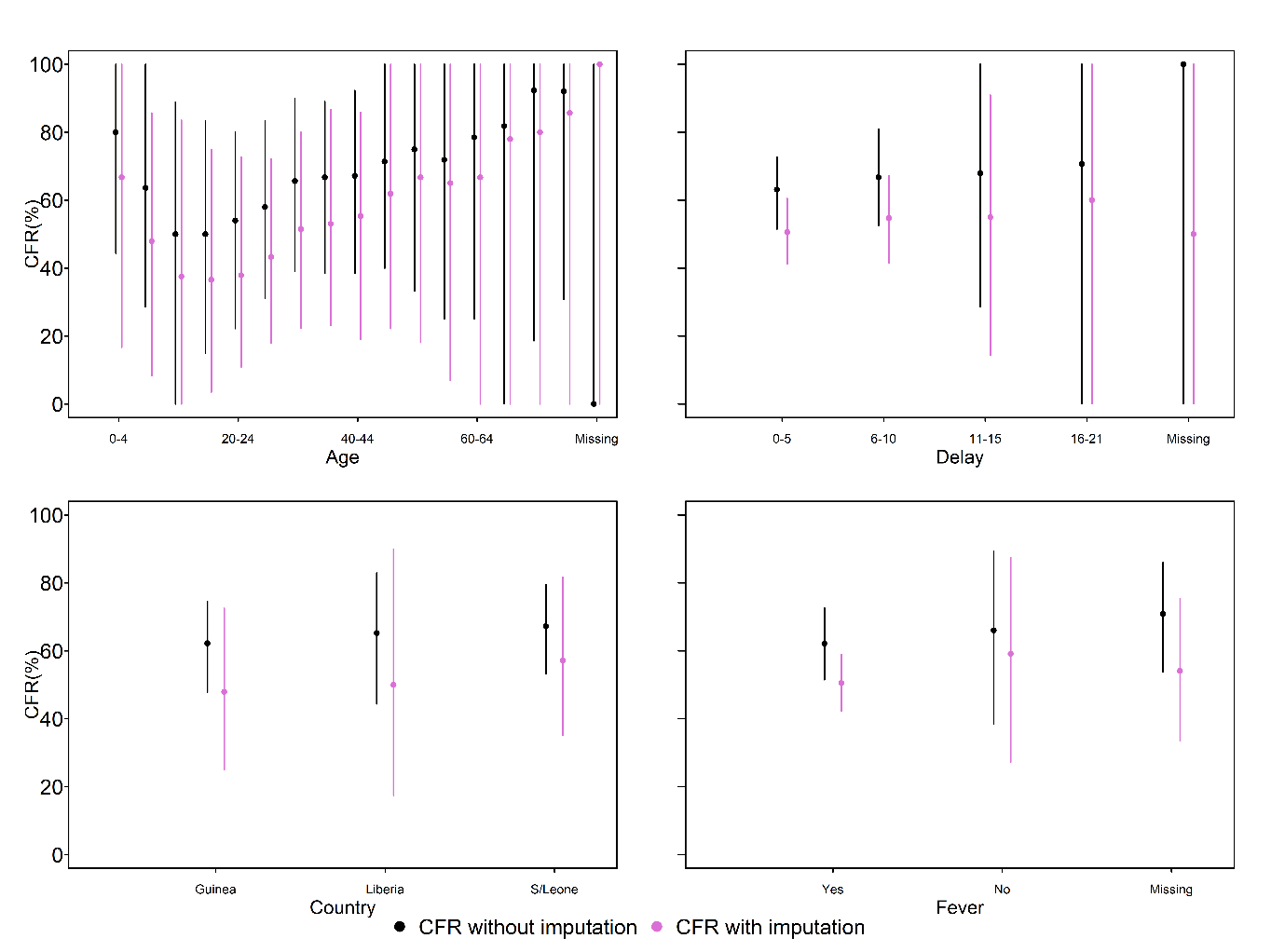


Figure S14: CFR estimates without (black) and with (orchid) imputation for age, delay, country, and fever, for confirmed cases for the validation data obtained using the (simplified) BRT model with tree complexity =27, learning rate=0.001, bag fraction=0.75, data partitioning ratio=0.65. Median and 95% confidence intervals (CI) plotted (based on 1,000 bootstrap realisations).

#### CFR for other Predictors


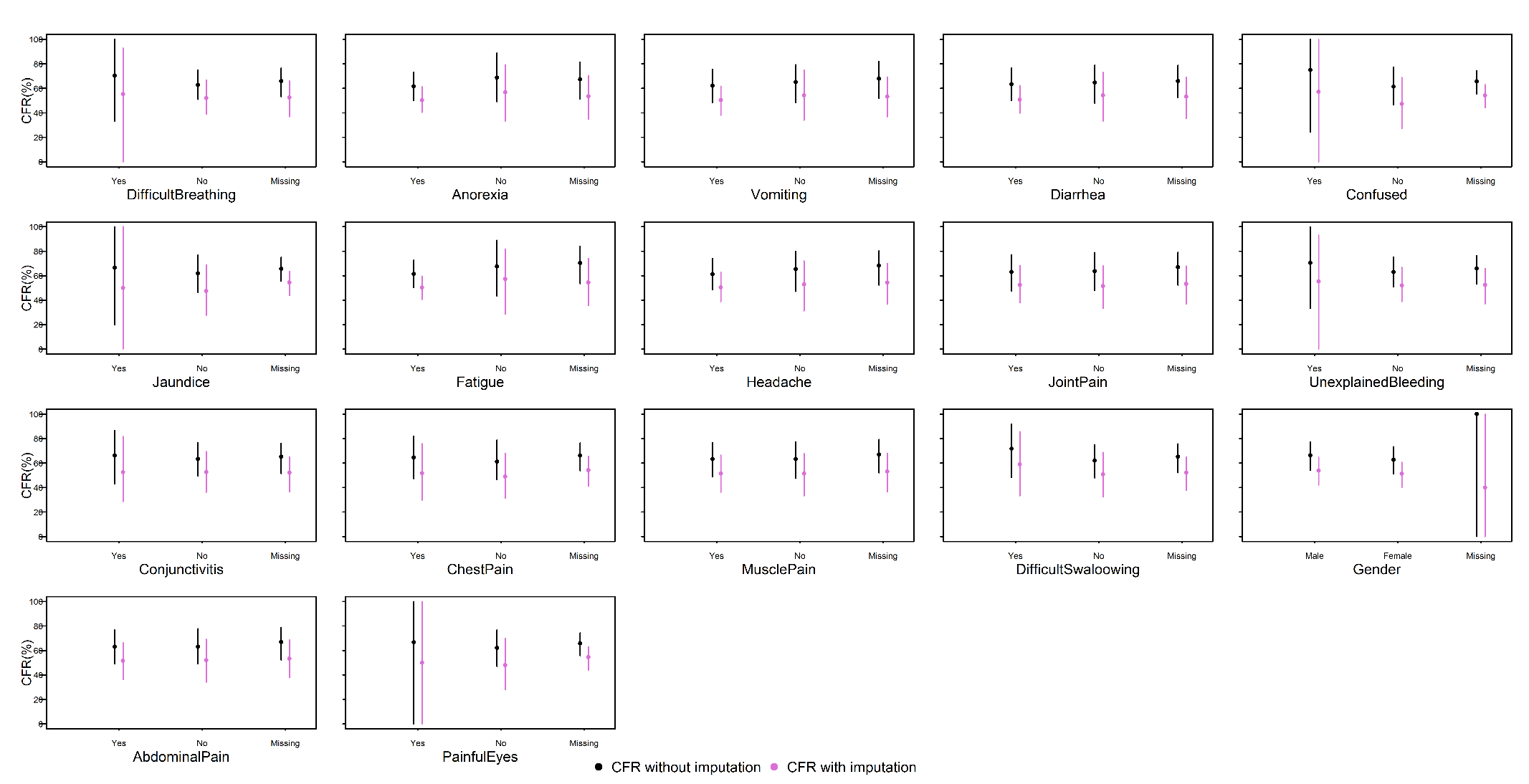


Figure S15: CFR estimates without (black) and with (orchid) imputation for other predictors, for confirmed probable cases obtained for the validation data using the (simplified) BRT model with tree complexity =10, learning rate=0.01, bag fraction=0.5, data partitioning ratio=0.8. Median and 95% confidence intervals (CI) plotted (based on 1,000 bootstrap realisations).

For example, for confirmed, probable and suspected cases, the performance of the model on the training data (e.g. AUC= 93.2 (95% CI: 87.1-98.3) is higher than that on the out-of-sample data (e.g. AUC= 76.2 (95% CI: 56.3-82.3). This is demonstrated by the comparison of the observed CFR with the CFR of the training data and the imputed CFR. To improve the CFR estimates and make the estimates more representative, we used two approaches to reduce the bias. These were (i) we combined the imputed CFR with the observed CFR to estimate CFR with imputation and (ii) considering the out-of-sample performance (i.e. sensitivity and specificity) of the (BRT) imputation model, we estimated the adjusted CFR. Subsequently, in the main manuscript and the supplementary information, we report the CFR without imputation, CFR with imputation and the adjusted CFR with imputation.

## 2.9 CFR adjusted with imputation by gender and age (categories)

Gender, fever, and age are common predictors of epidemiological outcomes.[10] Gender, fever and age were significant predictors of EVD survival, however, this contradicts an earlier analysis of fever [11], this contradiction could be explained by differences in the sample size and by a selection of the subset of data included in the analysis. Furthermore, this could also be indicative of an overwhelmed system earlier in the outbreak where many people were hospitalised as a precaution. Estimates adjusted with imputation are presented in Table S7.

Table S7: CFR estimates with imputation and confidence intervals are calculated from 1000 bootstraps of a BRT model. The BRT parameters used for imputation are tc=27, data partitioning=0.65, lr=0.001 and bf=0.75.

| Predictors | CFR adjusted with imputation | | |
| --- | --- | --- | --- |
|  | Confirmed cases  Median (95% CI) | Confirmed and probable cases Median (95% CI) | Confirmed, probable and suspected cases  Median (95% CI) |
| Gender | | | |
| Male | 67.6 (43.4-79.8) | 72.6 (43.1-81.8) | 84.8 (46.9-86.9) |
| Female | 59.2 (40.1-76.5) | 65.1 (40.5-80.2) | 79.8 (44.7- 84.9) |
| Age categories (years) | | | |
| 0_4 | 83.3 (49.8-91.5) | 88.3 (52.3-93.8) | 95.5 (69.0-97.5) |
| 5_9 | 67.9 (34.4 82.7) | 72.2 (33.5-84.9) | 82.6 (42.3-88.3) |
| 10_14 | 48.4 (23.7-78.2) | 53.3 (22.9-81.9) | 65.1 (28.1-84.3) |
| 15_19 | 41.3 (26.0-71.6) | 47.8 (26.9-74.5) | 63.7 (30.1-78.4) |
| 20_24 | 42.6 (28.8 74.1) | 47.0 (30.7 76.1) | 69.6 (32.9-81.1) |
| 25_29 | 46.7 (33.9-75.5) | 54.9 (34.5-77.9) | 71.6 (35.6-82.2) |
| 30_34 | 61.2 (41.2-80.8) | 65.6 (40.2-81.9) | 77.0 (41.0-85.2) |
| 35_39 | 57.1 (40.3-82.3) | 61.8 (41.3-83.5) | 78.0 (42.6-86.3) |
| 40_44 | 66.5 (40.3 81.7) | 72.4 (40.6-84.4) | 81.1 (43.4-87.3) |
| 45_49 | 72.8 (43.9-85.2) | 77.1 (44.3-86.5) | 82.7 (44.8-88.8) |
| 50_54 | 71.8 (45.0-85.9) | 79.0 (46.8-88.0) | 85.3 (49.2-90.7) |
| 55_59 | 76.8 (45.2 87.8) | 81.2 (46.4-90.5) | 85.6 (45.9-91.0) |
| 60_64 | 78.5 (48.8-89.5) | 81.9 (48.9-91.3) | 87.0 (54.3-92.5) |
| 65_69 | 82.2 (42.7-94.1) | 86.7 (43.4-95.1) | 90.3 (54.8-95.1) |
| 70_74 | 83.9 (55.2-95.8) | 87.9 (55.3-96.3) | 91.7 (63.4-97.7) |
| 75+ | 89.6 (58.6-98.0) | 91.0 (58.4-98.1) | 95.1 (69.8-97.8) |

|  |  |  |  |
| --- | --- | --- | --- |

## 2.10 CFR estimates without, unadjusted and adjusted with imputation for other predictors in simplified model

For predictors not included in the manuscript, figures S16 –S18 show the CFR estimates obtained without, unadjusted with imputation and adjusted with imputation ‘confirmed, probable and suspected’, ‘confirmed and probable’ and ‘confirmed’ cases, respectively. We found that the confidence intervals overlap and the confidence intervals for the CFR unadjusted with imputation and the adjusted CFR are wider. However, because imputed CFR include all cases and the adjusted CFR account for sensitivity and specificity of the BRT model, the CFR estimated using imputation are more representative and should be closer to the ‘true’ CFR during the epidemic.

### 2.8.1 Confirmed, probable and suspected cases


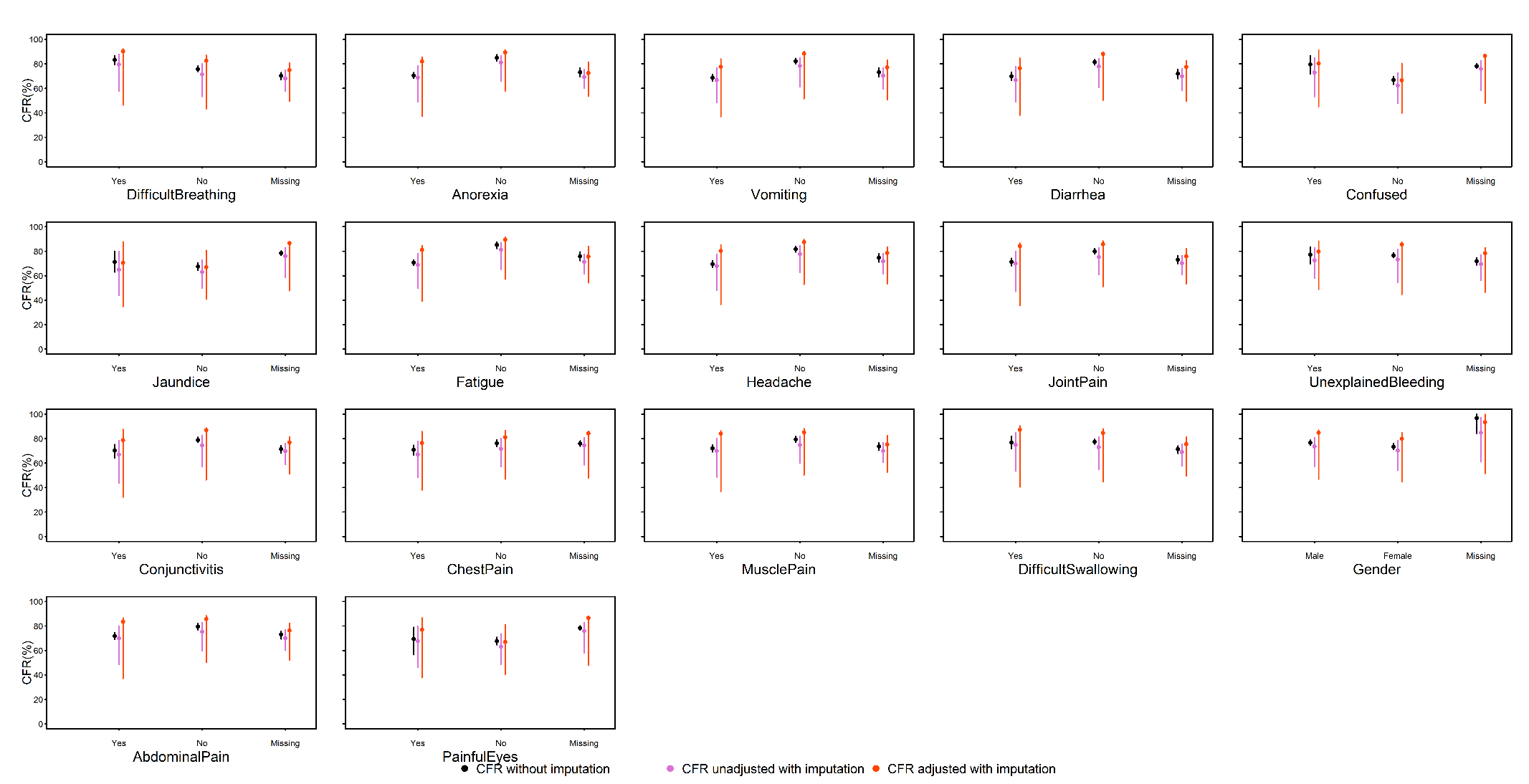


Figure S16: CFR for the other predictors in the simplified model without imputation (black), with imputation unadjusted(orchid) and with imputation adjusted (orange) for estimated BRT sensitivity and specificity. Median and 95% confidence intervals (CI) plotted (based on 1000 bootstrap realisations for ‘confirmed, probable and suspected’ cases).

### 2.8.2 Confirmed and probable cases


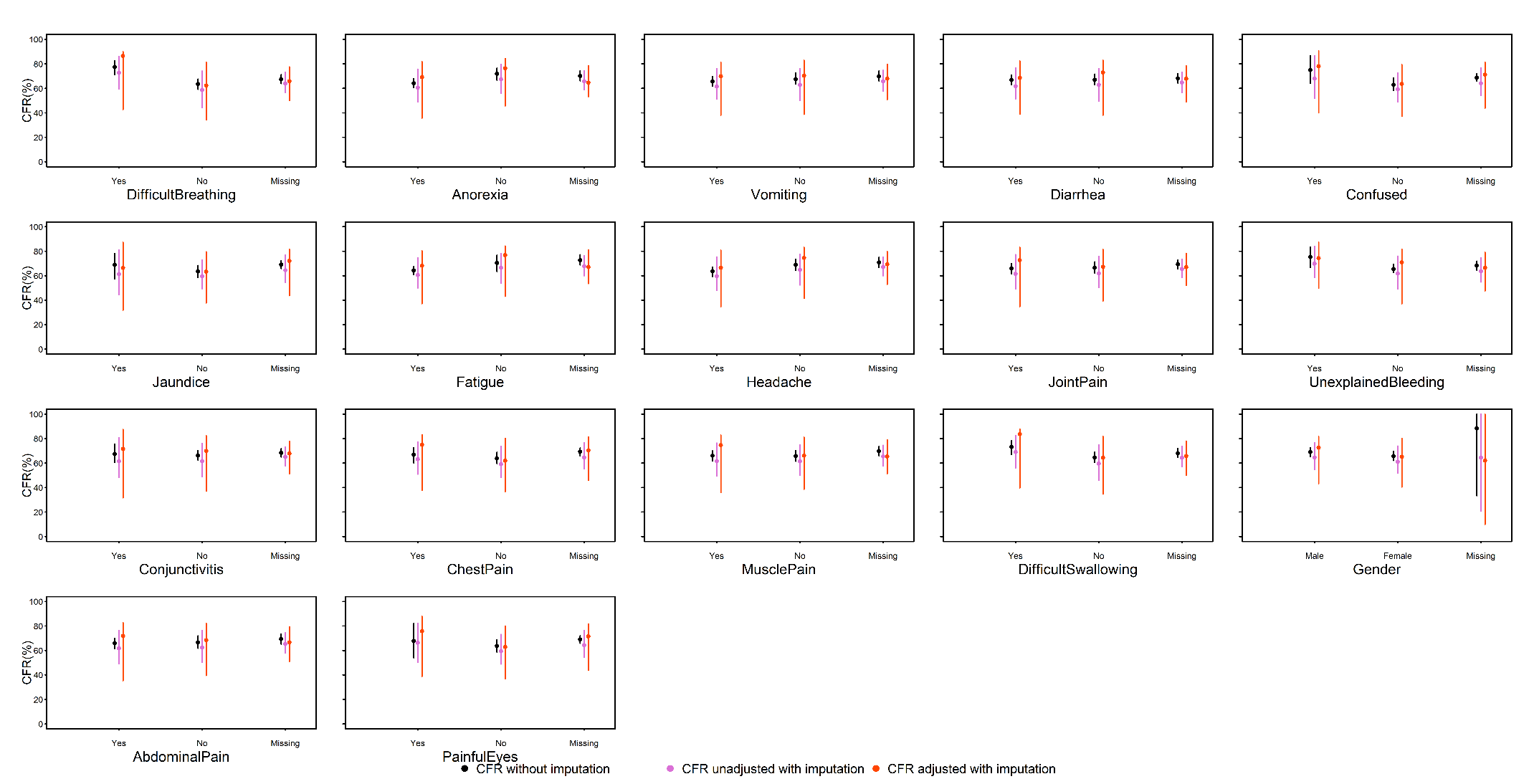


Figure S17: CFR for the other predictors in the simplified model without imputation (black), with imputation unadjusted (orchid) and with imputation adjusted (orange) for estimated BRT sensitivity and specificity. Median and 95% confidence intervals (CI) plotted (based on 1000 bootstrap realisations for ‘confirmed and probable’ cases).

### 2.8.3 Confirmed cases


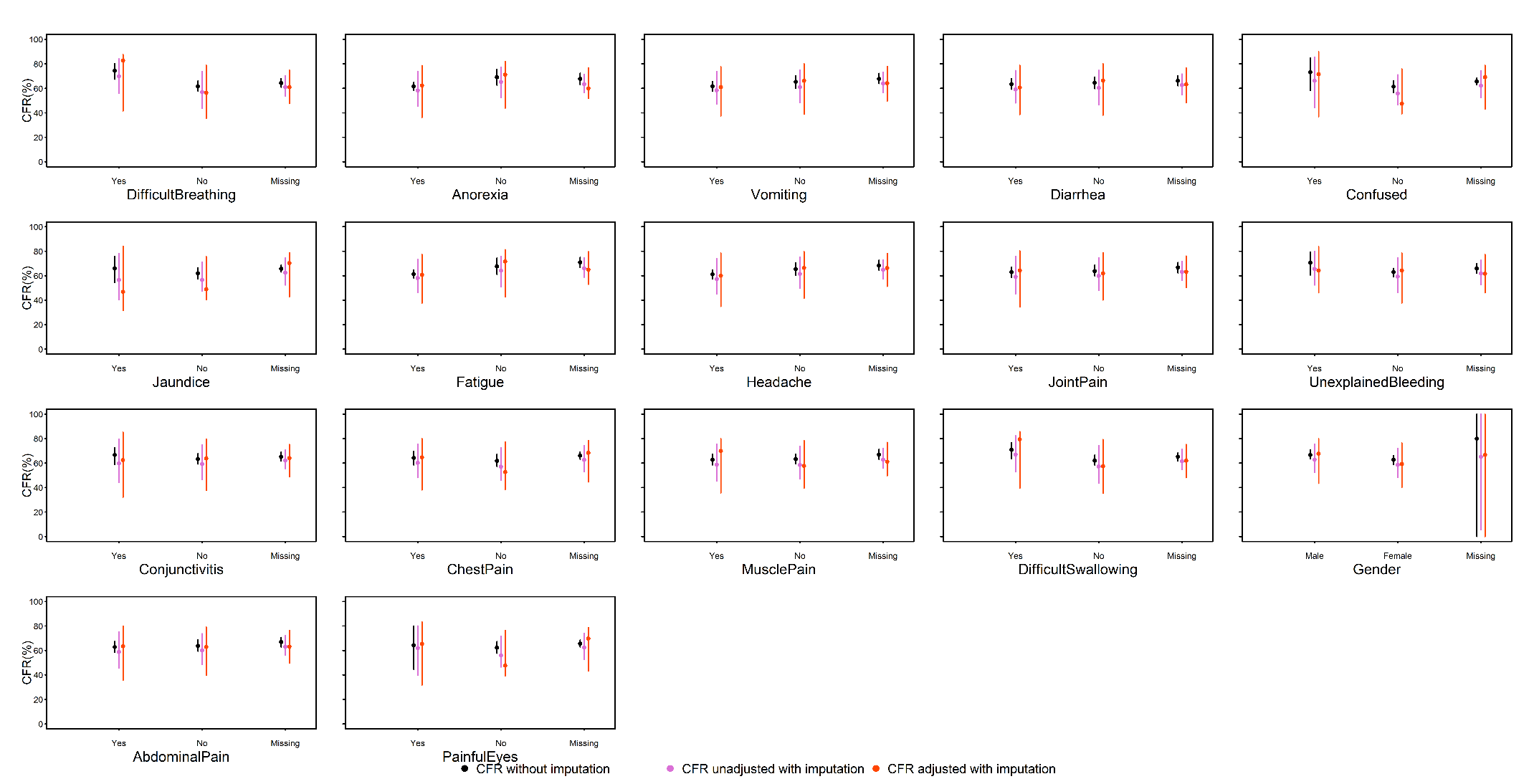


Figure S18: CFR for the other predictors in the simplified model without imputation (black), with imputation unadjusted (orchid) and with imputation adjusted (orange) for estimated BRT sensitivity and specificity. Median and 95% confidence intervals (CI) plotted (based on 1000 bootstrap realisations for ‘confirmed’).

## 2.11 Sampling to explore simulated missingness

Although the adjusted CFR estimates with imputation corroborate with estimates reported in the literature, they were relatively higher than those estimated unadjusted with imputation. While CFR unadjusted with imputation reflects the bias in the BRT model, adjusted CFR estimates account for this bias are therefore worth reporting. To further validate these adjusted CFR estimates, we carried out a simulated missingness analysis (detailed algorithm described in section 1.9). Briefly, if we know the CFR of a dataset but pretend as if this CFR is unknown, using the sensitivity and specificity of the BRT model and the tp function, the re-estimated adjusted CFR should be similar to the known CFR. Figure S19 shows that for different case combinations, CFR for imputed cases are similar to that for simulated missingness. For instance, for confirmed, probable and suspected cases, CFR for imputed cases was 66.1% (95% CI: 22.7%-88.2%) and CFR for simulated missingness was 65.0% (95% CI: 14.9%-89.2%) . Perhaps also, the inclusion of probable and suspected cases which lack laboratory confirmation of EVD is driving the higher adjusted CFR estimates. For instance, for laboratory confirmed cases, the overall adjusted CFR estimate is very similar to the CFR estimated by Garske and colleagues [12]. The adjusted overall CFR we estimated was 62.0% (95% CI: 51.6%-72.5%) while that estimated by Garske and colleagues was 62.9% (95% CI: 61.9%-64.0%) [12].


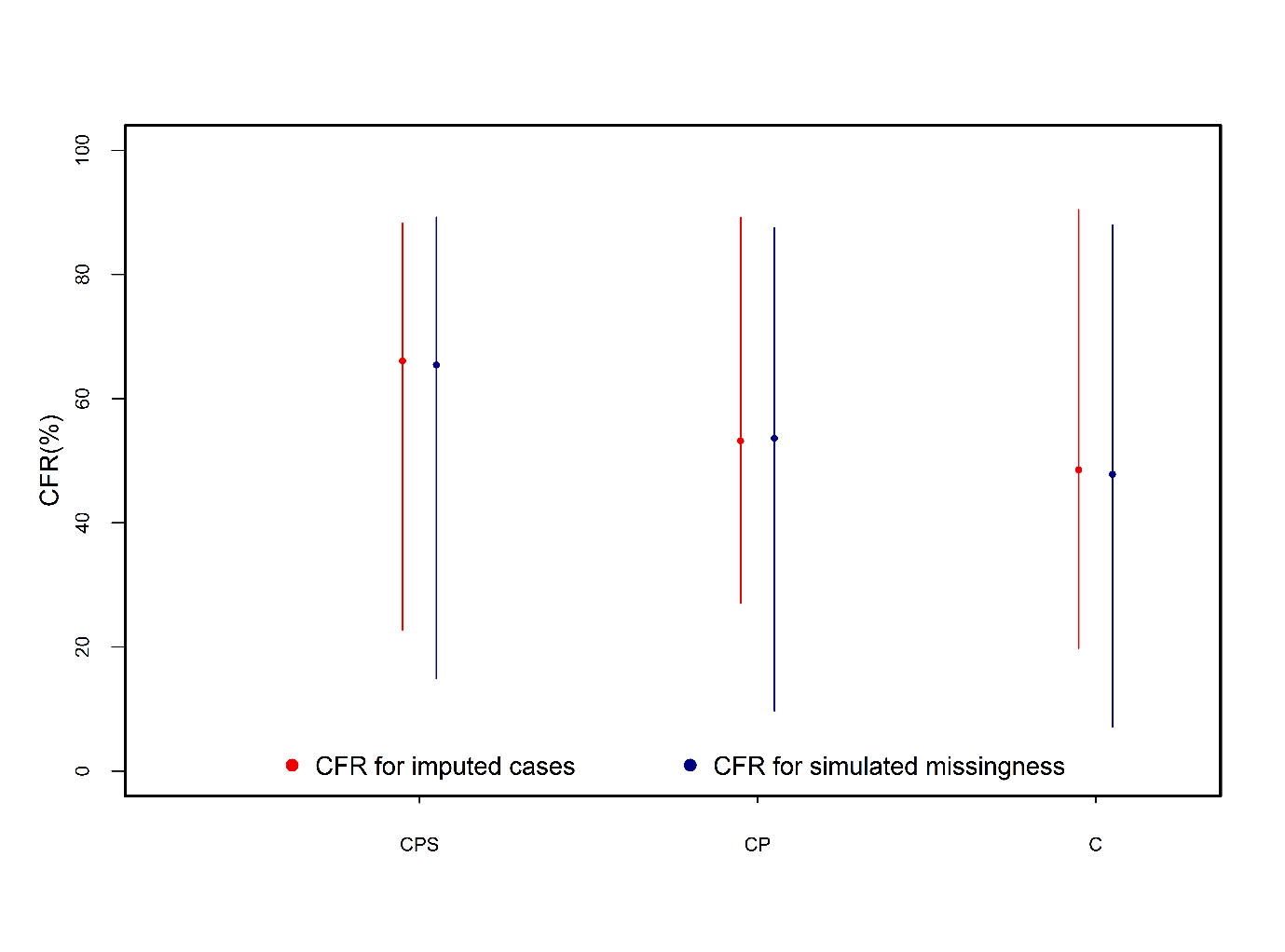


Figure S19: Imputed CFR and simulated missingness CFR for CPS (i.e. confirmed, probable and suspected) cases, CP (i.e. confirmed and probable) cases and C (i.e. confirmed) cases.

# 3 Sensitivity Analysis

## 3.1 Comparison of known and unknown outcome data for ‘confirmed’ and ‘confirmed and probable’ cases

Figure S20 shows the proportion (i.e. dead, alive and missing) of EVD cases by age and reporting-delay for ‘confirmed and probable’ and ‘confirmed’ cases.


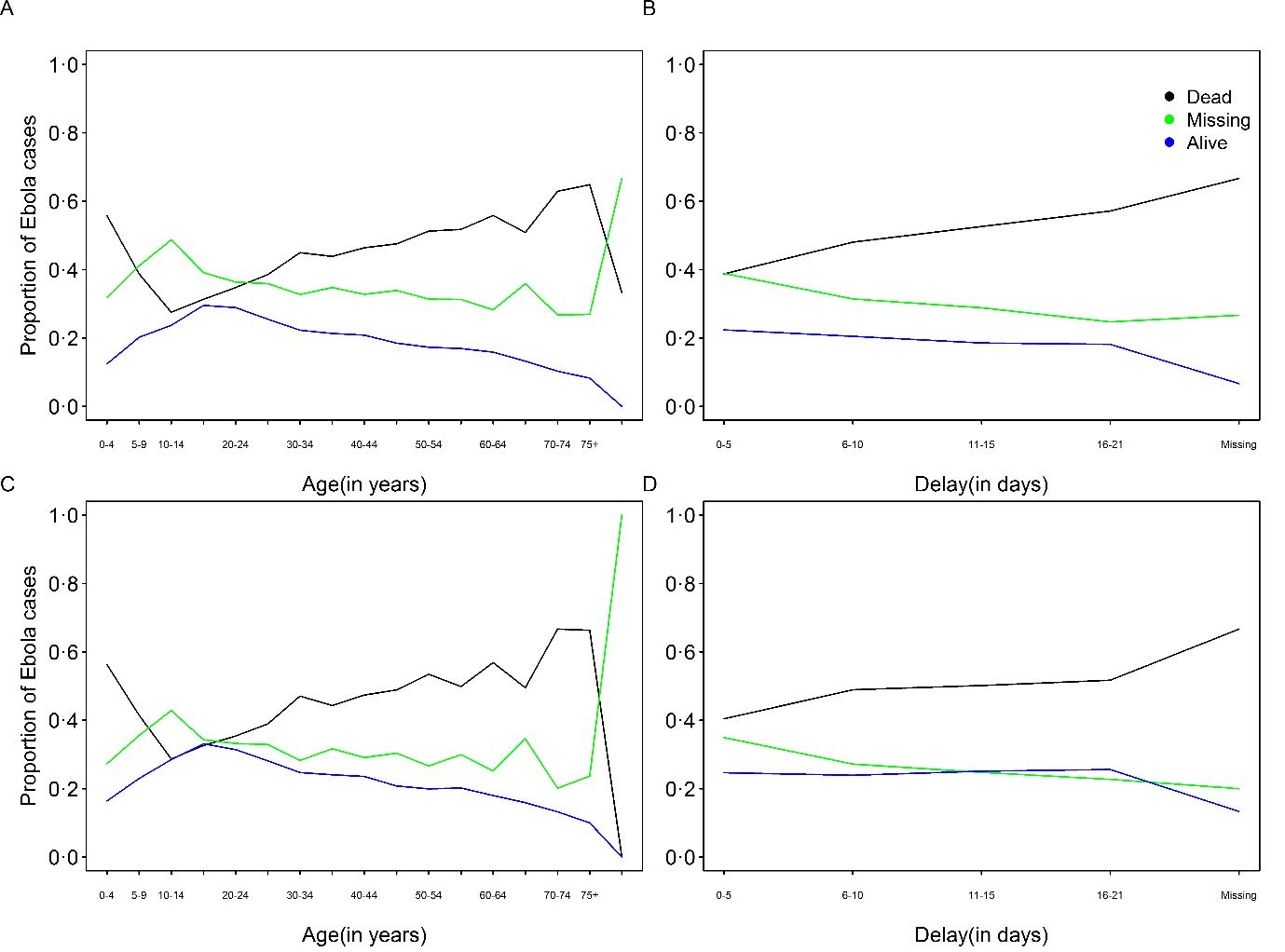


Figure S20: A, B: EVD case proportions for confirmed and probable cases by age and reporting-delay. C, D: EVD case proportion for confirmed cases by age and reporting-delay.

## 3.2 BRT model parameterisation for confirmed case data

Using non-bootstrap resampling (i.e. 50 realisations without replacement for the 72 hyperparameter combinations), re-parameterising the model for ‘confirmed’ case resulted in similar out-of-sample predictive performance (sensitivity=70% and AUC=78%) (Figure S22) compared to that for confirmed, probable and suspected EVD cases (sensitivity=70% and AUC=78%) (Figure S1) and the hyperparameters at which this performance was achieved remained unchanged: tc=27, lr=0.001, bf=0.75 and data partitioning ratio=0.65.


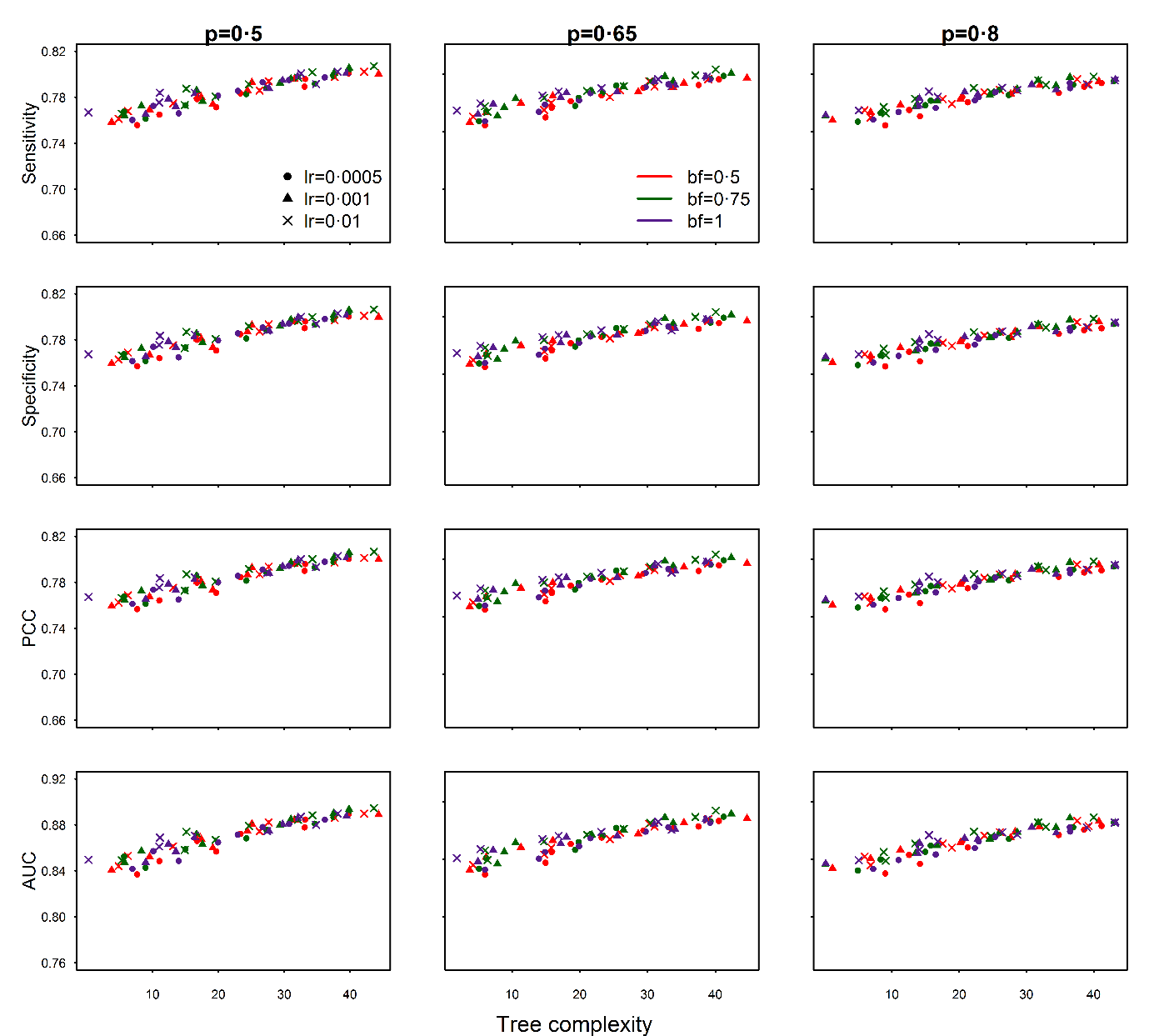


Figure S21: Goodness-of-fit accuracy for confirmed cases. p is the proportion of data used as training set. p=0.5, p=0.65 and p=0.8, respectively. PCC=Percentage Correctly Classified, AUC area under the receiver operating characteristic (ROC) curve. This shows an optimistic training performance but also indicates that we are not overfitting the model.


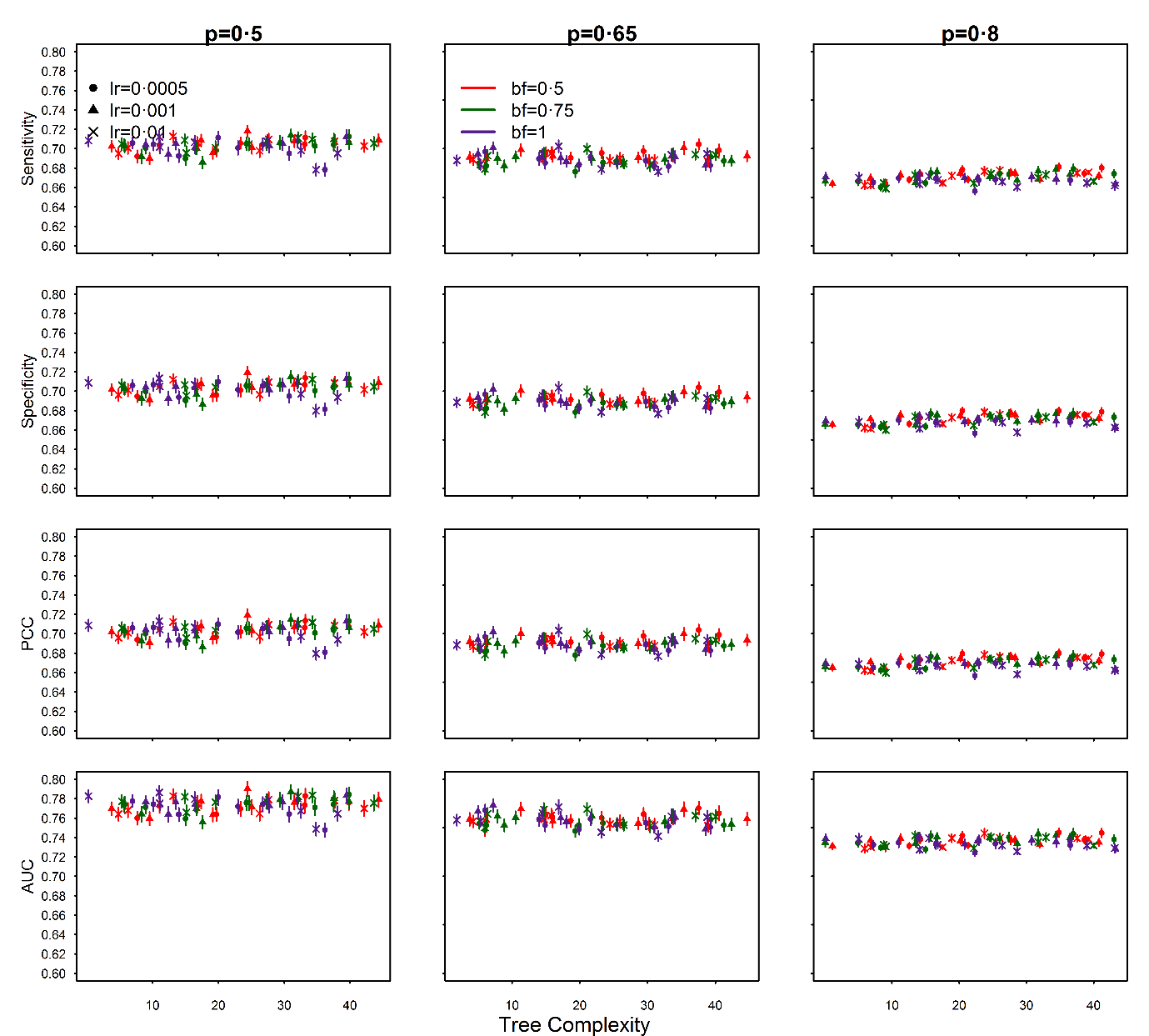


Figure S22: Out-of-sample accuracy for confirmed and probable cases. p is the proportion of data used as training set. p=0.5, p=0.65 and p=0.8, respectively. PCC=Percentage Correctly Classified, AUC area under the receiver operating characteristic (ROC) curve. This shows, an overall lower performance for confirmed only data compared to confirmed, probable and suspected data. The hyperparameters broadly remain the same as for confirmed, probable and suspected case data.

## 3.3 Minimal model predictors for variation in case definition

**Confirmed and probable cases**

We report the important predictors in the model simplified for confirmed and probable cases and for confirmed cases.

Table S8: Relative contributions (%) of the important predictors used in BRT models using 10-fold cross-validation, tc = 27, lr = 0.001 and bf = 0.75, for confirmed and probable cases, and trained on 1000 training sets generated by randomly sampling 65% cases with known survival outcomes . The minimal model used 10 predictors.

| Predictors | Relative Contribution (%) |
| --- | --- |
| District | 42.2 |
| Hospitalisation status | 34.3 |
| Age | 16.1 |
| Difficult Breathing | 2.5 |
| Delay | 1.4 |
| Quarter | 1.4 |
| Anorexia | 0.8 |
| Case classification | 0.6 |
| Fever | 0.3 |
| Fatigue | 0.3 |

**Confirmed cases**

Table S9: Relative contributions (%) of the important predictors used in BRT models using 10-fold cross-validation, tc = 27, lr = 0.001 and bf = 0.75, for confirmed cases, and trained on 1000 training sets generated by randomly sampling 65% cases with known survival outcomes . The minimal model used 10 predictors.

| Predictors | Relative Contribution (%) |
| --- | --- |
| District | 43.8 |
| Hospitalisation status | 37.2 |
| Age | 14.8 |
| Quarter | 1.4 |
| Difficult Breathing | 1.1 |
| Anorexia | 0.5 |
| Fever | 0.5 |
| Delay | 0.4 |
| Fatigue | 0.3 |
| Case classification | - |

## 3.4 CFRs for variations in case definition

In Table S10, S11 we show the CFR estimates, without imputation, unadjusted with imputation, and adjusted with imputation for ‘confirmed and probable cases’ and ‘confirmed cases’

Table S10: CFR for confirmed cases. Overall and country-level estimates. All CFR estimates and corresponding CIs were calculated using a non-parametric bootstrap of the BRT model.

|  | Cases for dataset without imputation | Cases for dataset with imputation | CFR without imputation  (95% CI) | Unadjusted CFR with imputation  (95% CI) | Adjusted CFR with imputation  (95% CI) |
| --- | --- | --- | --- | --- | --- |
| Guinea | 3,297 | 3,304 | 62.1 (58.4-66.0) | 62.0 (58.4-66.1) | 62.0 (58.7-66.0) |
| Liberia | 2,384 | 3,743 | 65.1 (59.0-70.2) | 63.2 (53.9-72.4) | 67.0 (47.0-75.6) |
| Sierra Leone | 3,790 | 9,397 | 67.0 (63.6 -71.4) | 58.8 (43.4-80.2) | 61.1 (30.8-86.1) |
| Overall* | 9,471 | 16,444 | 64.6 (62.4-67.2) | 60.4 (50.5-73.4) | 62.5 (42.6-77.7) |

Table S11: CFR for confirmed and probable cases. Overall and country-level estimates. All CFR estimates and corresponding CIs were calculated using a non-parametric bootstrap of the BRT model.

|  | Cases for dataset without imputation | Cases for dataset with imputation | CFR without imputation  (95% CI) | Unadjusted CFR with imputation  (95% CI) | Adjusted CFR with imputation  (95% CI) |
| --- | --- | --- | --- | --- | --- |
| Guinea | 3,740 | 3,747 | 65.9 (62.3-69.7) | 65.8 (62.4-69.7) | 65.7 (62.3-69.7) |
| Liberia | 3,131 | 5,343 | 66.7 (62.2-71.9) | 66.4 (56.2-76.1) | 77.2 (42.7-80.9) |
| Sierra Leone | 4,421 | 11,264 | 69.0 (65.1-73.1) | 59.4 (44.7-80.5) | 62.5 (30.2-87.1) |
| Overall* | 11,292 | 20,354 | 67.3 (64.8-70.1) | 62.6 (53.5-75.4) | 68.5 (42.2-80.5) |

3.5 BRT imputation performance for variations in case definition

Out-of-sample performance obtained using the final (simplified) BRT model for ‘confirmed and probable’ and ‘confirmed’ cases (Table S12).

Table S12: BRT Imputation performance (simplified model) for confirmed cases and for confirmed and probable cases.

| Performance Measures | Imputation Performance | |
| --- | --- | --- |
|  | Confirmed Cases Model Performance  Median (%) (95% CI) | Confirmed and Probable Cases Model Performance  Median (%) (95% CI) |
| Sensitivity | 58.8 (47.4-67.4) | 60.0 (48.0-69.2) |
| Specificity | 58.8 (48.3-67.4) | 59.9 (48.1.8-68.8) |
| PCC | 58.8 (48.3-67.0) | 59.9 (48.3-68.5) |
| AUC | 63.2 (51.6-72.5) | 64.5 (52.6-74.7) |

##

## 3.6 CFR estimates by age group, delay, country and fever for other case definitions.

We show the CFR estimates without, unadjusted with imputation and adjusted with imputation obtained with the final (simplified) BRT model for ‘confirmed and probable’ and ‘confirmed’ cases (Figures S23, S24). The CFR adjusted CFR with imputation for confirmed and probable cases is consistent with CFR estimates for confirmed and probable cases in the present outbreak in the Democratic Republic of Congo (DR Congo), whereas, the adjusted CFR with imputation for confirmed cases is consistent with CFR cases for the West African Ebola epidemic [12-14]. For instance, Garske and colleagues estimated CFR for confirmed cases as 62.9% (95% CI: 61.9%-64.0%) while the adjusted CFR we estimated for confirmed cases was 62.5% (95% CI: 42.6%-77.7%). As of 26 December 2018, the CFR for confirmed and probable cases in DR Congo was 60.4% (95% CI: 56.3%-64.4%) while the overall adjusted CFR with imputation for West Africa we estimated was 68.5% (95% CI: 42.2%-80.0%) [15]. However, on a note of caution, the CFR for the DR Congo outbreak is subject to change due to ongoing reclassification, retrospective investigation, and the ongoing availability of confirmed laboratory results.

### 3.6.1 Confirmed and probable cases


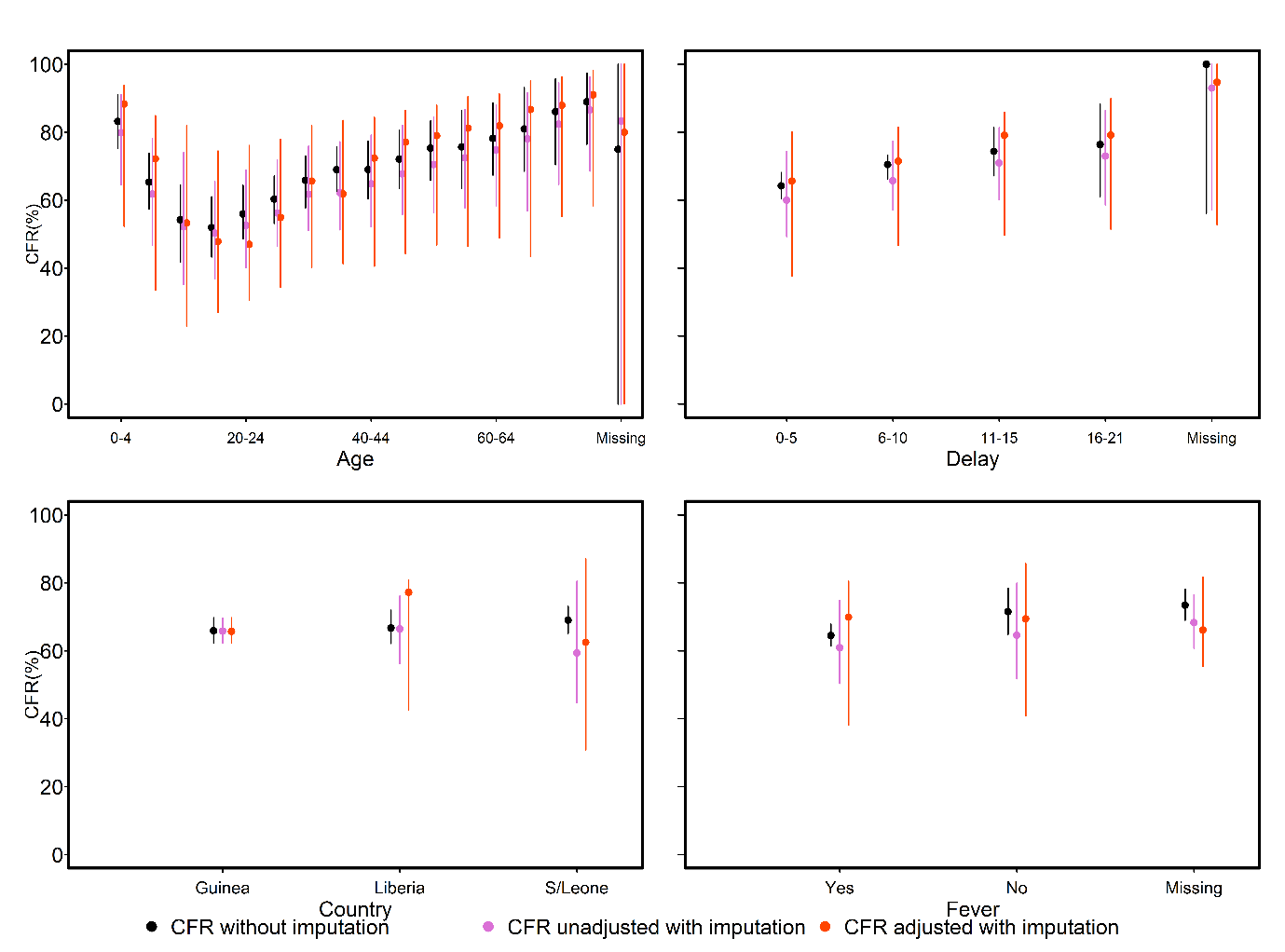


Figure S23: CFR by age group, delay, country, and fever without imputation (black), with imputation unadjusted (orchid) and with imputation adjusted (orange) for BRT sensitivity and specificity. Median and 95% confidence intervals (CI) plotted (based on1000 bootstrap realisations for ‘confirmed and probable’ cases).

### 3.6.2 Confirmed cases


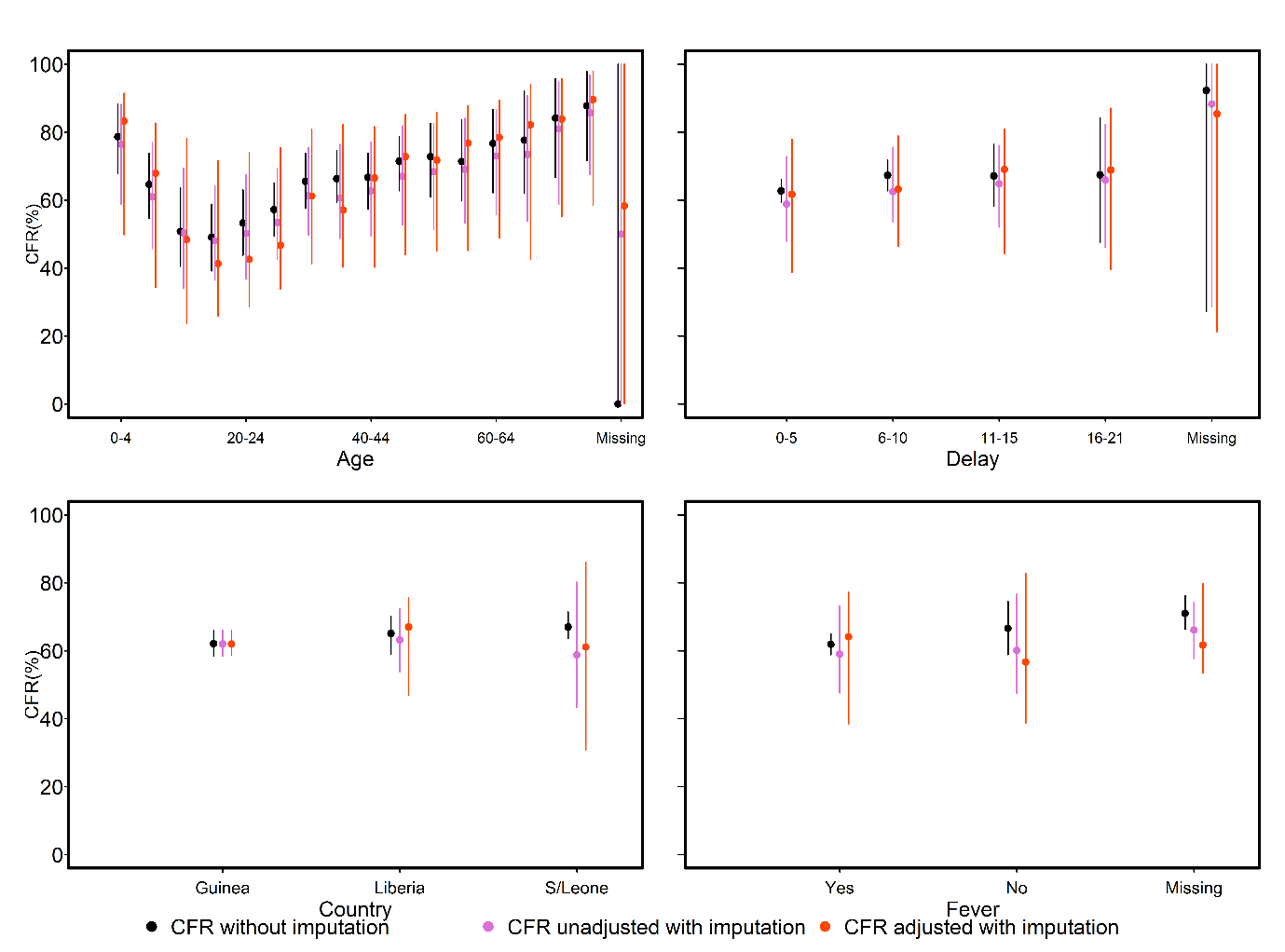


Figure S24: CFR by age group, delay, country, and fever without imputation (black), with imputation (orchid) unadjusted and with imputation adjusted (orange) for BRT sensitivity and specificity. Median and 95% confidence intervals (CI) plotted (based on 1000 bootstrap realisations for ‘confirmed and probable’ cases).

# 4 References

1. WHO Ebola Response Team. Ebola virus disease in West Africa—the first 9 months of the epidemic and forward projections. N Engl J Med **2014**; 2014(371): 1481-95.

2. Bhatt S, et al. The global distribution and burden of dengue. Nature **2013**; 496(7446): 504-7.

3. Friedman J, Hastie T, Tibshirani R. The elements of statistical learning: Springer series in statistics New York, **2001**.

4. Elith J, Leathwick JR, Hastie T. A working guide to boosted regression trees. J Anim Ecol **2008**; 77(4): 802-13.

5. Kohavi R. A Study of Cross-Validation and Bootstrap for Accuracy Estimation and Model Selection. International Joint Conference on Artificial Intelligence **1995**; 14(12): 1137-43.

6. Vanneschi L, Farinaccio A, Mauri G, Antoniotti M, Provero P, Giacobini M. A comparison of machine learning techniques for survival prediction in breast cancer. BioData Mining **2011**; 4(1): 12-.

7. James G, Witten D, Hastie T, Tibshirani R. An introduction to statistical learning: Springer, **2013**.

8. Sergeant E. Package ‘ RSurveillance ’. **2016**.

9. WHO Ebola Response Team. West African Ebola epidemic after one year—slowing but not yet under control. N Engl J Med **2015**; 372(6): 584-7.

10. Michel G, Bisegger C, Fuhr DC, Abel T. Age and gender differences in health-related quality of life of children and adolescents in Europe: a multilevel analysis. Qual Life Res **2009**; 18(9): 1147.

11. WHO Ebola Response Team. Ebola virus disease among male and female persons in West Africa. N Engl J Med **2016**; 374(1): 96-8.

12. Garske T, Cori A, Ariyarajah A, et al. Heterogeneities in the case fatality ratio in the West African Ebola outbreak 2013–2016. Phil Trans R Soc B **2017**; 372(1721): 20160308.

13. Barry A, Ahuka-Mundeke S, Ahmed YA, et al. Outbreak of Ebola virus disease in the Democratic Republic of the Congo, April–May, 2018: an epidemiological study. The Lancet **2018**; 392(10143): 213-21.

14. Focosi D, Maggi F. Estimates of Ebola virus case-fatality ratio in the 2014 West African outbreak. Clin Infect Dis **2015**; 60(5): 829-.

15. World Health Organisation. Ebola External Situation Report 21. Available at: <http://apps.who.int/iris/bitstream/handle/10665/277405/SITREP-EVD-DRC-20181227-eng.pdf?ua=1>. Accessed 04 January 2019.
